# Antifungal Tc17 cells are durable and stable, persisting as long-lasting vaccine memory without plasticity towards IFNγ cells

**DOI:** 10.1371/journal.ppat.1006356

**Published:** 2017-05-22

**Authors:** Som Gowda Nanjappa, Andrew J. McDermott, J. Scott Fites, Kevin Galles, Marcel Wüthrich, George S. Deepe, Bruce S. Klein

**Affiliations:** 1 Department of Pediatrics, University of Wisconsin School of Medicine and Public Health, Madison, WI, United States of America; 2 Department of Internal Medicine, Division of Infectious Diseases, University of Cincinnati, College of Medicine, Cincinnati, OH, United States of America; 3 Department of Internal Medicine, University of Wisconsin School of Medicine and Public Health, Madison, WI, United States of America; 4 Department of Medical Microbiology and Immunology, University of Wisconsin School of Medicine and Public Health, Madison, WI, United States of America; Geisel School of Medicine at Dartmouth, UNITED STATES

## Abstract

Our understanding of persistence and plasticity of IL-17A^+^ memory T cells is clouded by conflicting results in models analyzing T helper 17 cells. We studied memory IL-17A^+^ CD8^+^ T-cell (Tc17) homeostasis, persistence and plasticity during fungal vaccine immunity. We report that vaccine-induced memory Tc17 cells persist with high fidelity to the type 17 phenotype. Tc17 cells persisted durably for a year as functional IL-17A^+^ memory cells without converting to IFNγ^+^ (Tc1) cells, although they produced multiple type I cytokines in the absence of residual vaccine antigen. Memory Tc17 cells were canonical CD8^+^ T cells with phenotypic features distinct from Tc1 cells, and were Ror(γ)t^hi^, TCF-1^hi^, T-bet^lo^ and EOMES^lo^. In investigating the bases of Tc17 persistence, we observed that memory Tc17 cells had much higher levels of basal homeostatic proliferation than did Tc1 cells. Conversely, memory Tc17 cells displayed lower levels of anti-apoptotic molecules Bcl-2 and Bcl-xL than Tc1 cells, yet were resistant to apoptosis. Tc1 cells required Bcl-2 for their survival, but Bcl-2 was dispensable for the maintenance of Tc17 cells. Tc17 and Tc1 cells displayed different requirements for HIF-1α during effector differentiation and sustenance and memory persistence. Thus, antifungal vaccination induces durable and stable memory Tc17 cells with distinct requirements for long-term persistence that distinguish them from memory Tc1 cells.

## Author summary

CD4^+^ T-cell deficient patients such as those with AIDS and idiopathic CD4^+^ T-cell lymphopenia are vulnerable to systemic fungal infections. We previously showed that CD8^+^ T cells can be exploited in CD4^+^ T cell deficient hosts for vaccine immunity against lethal fungal pneumonia in mice and that IL-17A production by these cells (Tc17) is essential. Existing dogma holds that IL-17A producing CD4^+^ T cells (Th17) are highly plastic, unstable, and convert into IFNγ producing cells, losing the capacity to produce IL-17A, which is the signature feature of Tc17 cells. Here, we show that vaccine-elicited antifungal Tc17 cells are maintained as stable and long-lasting memory cells that resist conversion into IFNγ cells (Tc1) and protect CD4^+^ T cell deficient hosts against lethal pulmonary fungal infection. Antifungal Tc17 cells displayed features that define classical memory cells. However, memory Tc17 exhibited different requirements than Tc1 cells in the factors that promote T cell survival, including anti-apoptotic molecules Bcl-2 and Bcl-xl, and HIF-1α, which aids survival of cells in lower oxygen conditions found during inflammation. Thus, our study reveals that fungal vaccination elicits a durable, stable population of Tc17 cells with distinct features of survival needed for preventing infection in immunocompromised hosts.

## Introduction

Memory Th1 and Tc1 (IFNγ^+^ CD8^+^ T cells) cells have been well characterized. Following the expansion phase of an immune response, a pool of memory precursor cells (~10%) survives the contraction phase, and slowly differentiates into long-lasting memory cells[1]. Memory Th1 and Tc1 cells are maintained stably for years, often lifelong, even in the absence of cognate antigen [2–4]. Maintenance of memory Th1 and Tc1 is chiefly regulated by IL-7 for survival and IL-15 for intermittent homeostatic turnover[5–7]. Type 1 memory precursors are CD127^hi^, KLRG-1^lo^, CD27^hi^, CD122^hi^, Bcl-2^hi^, T-bet^lo^, Eomes^hi^ and TCF-1^hi^[8,9], and the expression of CD62L and CCR7 distinguish “central memory” vs. “effector memory” T-cell subsets[10].

In contrast to Th1 and Tc1 cells, attributes of Th17 cells are less well understood and the persistence of *memory* Th17 cells is debated. *In vitro* polarized Th17 cells that are adoptively transferred persist and portray stem-cell like features[11–14]. Conversely, *in vivo* induction and persistence of Th17 cells depends on route and type of infection. Th17 cells induced upon mucosal *Listeria monocytogenes* infection are short-lived due to their CD27^lo^ phenotype[15,16], whereas subcutaneous injection of *Mycobacterium tuberculosis* antigens induces long-lasting Th1 and Th17 memory cells[16]. Acute cutaneous infection with *Candida albicans* induces Th17 cells that quickly “switch off” IL-17 production[17], while oropharyngeal infection leads to induction of Th17 cells that persist for at least weeks[18]. Th17 cells are known to persist for a longer time under chronic inflammatory conditions, implying a role for lingering antigen and inflammation[19].

Unlike Th1 and Th2 cells, Th17 cells are poised to be highly plastic, presumably due to their epigenetic instability[20–22]. *In vitro* polarized Th17 cells either convert to Th1 cells or express both Th1 and Th17 cytokines after adoptive transfer into mice, possibly due to deprivation of signals from polarizing cytokines[23]. Host immune status also may impact Th17 plasticity since lymphopenia promotes expansion of Th1 cells[24]. In some models, immunization of mice with adjuvanted antigen drives Th17 responses, but the cells fail to maintain their phenotype and convert into Th1 cells. For example, in EAE models, Th17 cells show plasticity towards IFNγ and GM-CSF production and augment severity of the disease[25,26]. Similarly, *Candida*-specific human memory Th17 cells co-express IFNγ, and staphylococcus-induced Th17 cells express IL-10 upon re-stimulation[27].

Similar to Th17 cells, Tc17 cells form a distinct subset with widely ranging responses implicated in immunity, immunopathology and systemic autoimmunity [28]. Effector Tc17 cells mediate potent anti-viral, anti-fungal, and anti-tumor activity[29–31]^,33^. Tc17 effectors also potentiate autoimmune actions of Th17 cells during EAE[32] and exacerbate other autoimmune disorders due to their ability to express multiple cytokines e.g. IL-22, GM-CSF, M-CSF, IFNγ and IL-3[28]. Like Th17 cells, *in vitro* polarized Tc17 show plasticity towards IFNγ production[33], and donor Tc17 cells become plastic “inflammatory” iTc17 in recipients with GVHD[34].

Vaccination in the absence of CD4^+^ T cells induces Tc17 cells that confer resistance against lethal experimental fungal pneumonia[30], a feature that can be translated to immune-compromised individuals with CD4^+^ T cell lymphopenia that are susceptible to opportunistic fungal infection[35]. While memory Tc17 cells are required in this type of immunity, the persistence and plasticity of these cells have not been investigated.

In this study, we investigated the persistence, fidelity, plasticity and functionality of memory Tc17 cells by using an experimental fungal vaccine that is effective against lethal pneumonia in CD4^+^ T cell deficient hosts. We found that antifungal memory Tc17 cells are durable and persist long term. Memory Tc17 cells also showed little conversion into IFNγ producing cells, although the cells expressed multiple cytokines. We also found that memory Tc17 cells retain expression of RORγt and portray a phenotypic profile distinct from memory Tc1 cells. Memory Tc17 cells displayed higher proliferative renewal than did Tc1 cells, and lower levels of the anti-apoptotic molecule Bcl-2, which was not required for maintenance of these Tc17 cells. Memory Tc17 cells required HIF-1α for their homeostasis, whereas memory Tc1 cells did not require this factor. Our work provides new insight into distinct features that characterize and promote persistent, stable anti-fungal Tc17 cells induced upon vaccination.

## Results

### Anti-fungal memory Tc17 cells are long-lived

**Fig 1:**
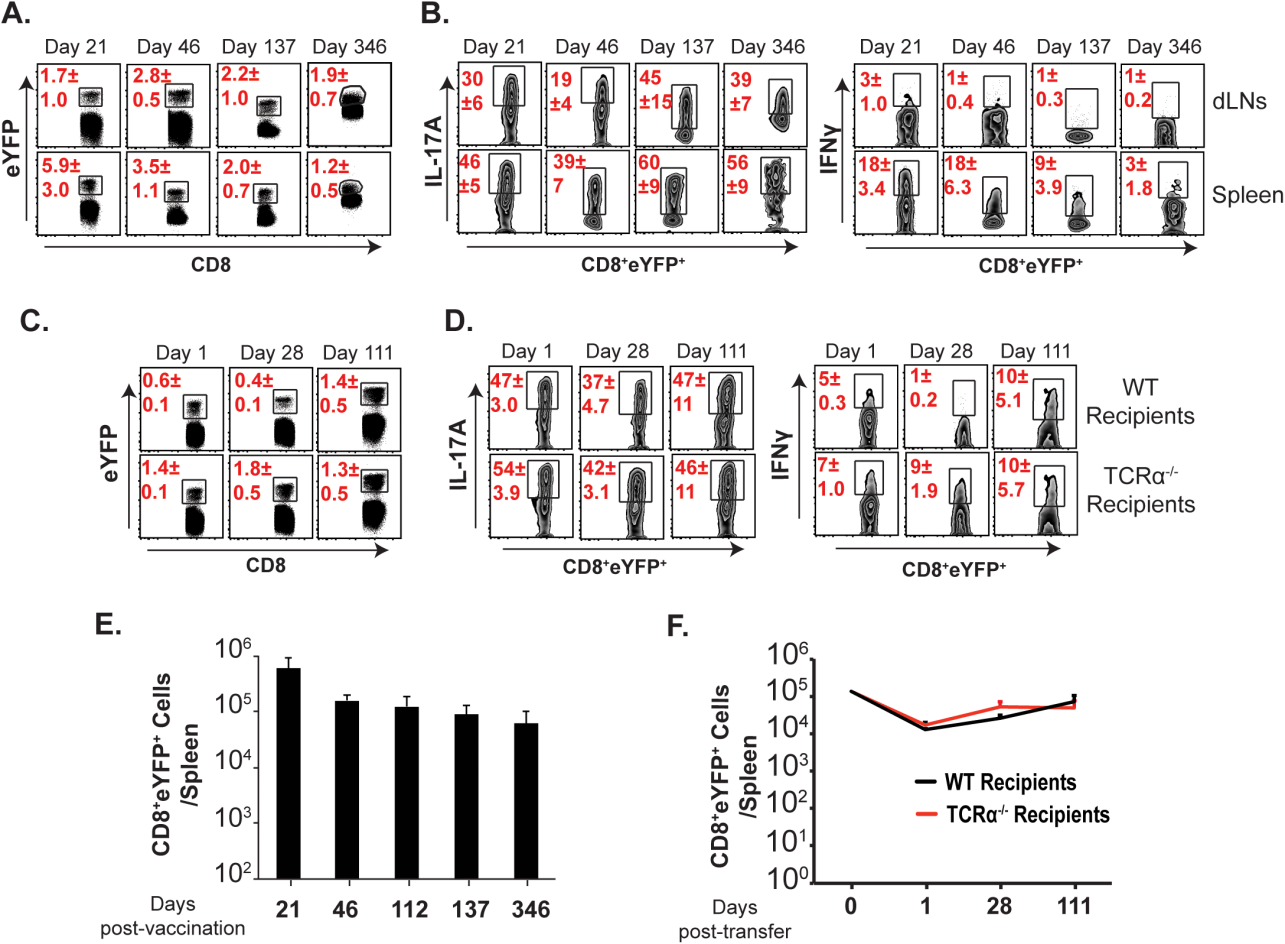
Long-term persistence, fidelity and plasticity of memory Tc17 cells. Naïve IL17a^Cre^R26R^eYFP^ mice were vaccinated with ~10^5^ CFU of #55 strain of B. *dermatitidis*. The draining lymph nodes (dLNs) and spleens were harvested on indicated days. Following staining, cells were analyzed by flow cytometry. Percent **(A)** and absolute numbers **(E)** of eYFP^+^ CD8^+^ T cells. Percent **(B)** cytokine producing cells among eYFP^+^ CD8^+^ T cells. N = 5 mice/group. **(C & F)** Percent and absolute numbers of eYFP^+^ cells in naïve WT or TCRα^-/-^ recipient mice in the spleens were analyzed by flow cytometry on indicated days following adoptive transfer of purified effector CD8^+^ T cells from IL17a^Cre^R26R^eYFP^ donor mice (day 23 post-vaccination, 12.7x10^4^ eYFP^+^ cells/mouse). Percent **(D)** cytokine producing cells among eYFP^+^ CD8^+^ T cells. N = 3–5 mice/group. Data is representative of at least two independent experiments. Mice were injected with GK1.5 throughout the experiment to deplete CD4^+^ T cells.

We first investigated the fate and longevity of memory Tc17 cells after vaccination. We used “fate-mapping” mice (IL-17A^cre^Rosa26^eYFP^)[17] where IL-17A-induced cells become indelibly marked with eYFP irrespective of the fate of IL-17A expression. Similar to a published report[17], ~30–50% of the eYFP^+^ cells expressed IL-17A (**S1A Fig**). Likewise, ~30–40% of total IL-17A^+^ cells also expressed eYFP[17]. To assess the persistence of memory Tc17 cells, we vaccinated IL-17A fate-mapping mice and enumerated the percent and absolute numbers of CD8^+^ eYFP^+^ cells in tissues (**Fig 1A & 1E; S1B Fig**). Effector CD8^+^ eYFP^+^ cells persisted as memory cells maintaining their phenotype up to 11 months post vaccination (**Fig 1A**). We previously noted that live vaccine yeast persist for ~7 weeks[36]. To exclude that persistent vaccine antigen accounts for the longevity of memory Tc17 cells, we purified effector CD8^+^ T cells and adoptively transferred them into naïve wild-type (WT) and TCRα^-/-^ recipient mice. At serial time points, we enumerated CD8^+^ eYFP^+^ T cells in draining lymph nodes (dLNs) and spleens (**Fig 1C & 1F; S1C Fig**). The intake of CD8^+^ eYFP^+^ cells was ~10% as expected (at day 1 post-transfer) and the remaining cells persisted stably even after 3 months rest in both immune-sufficient (WT) and -deficient (TCRα^-/-^) recipients (**Fig 1F**). Thus, antifungal memory Tc17 cells induced by vaccination persist stably even in the absence of vaccine antigen.

### Fidelity of anti-fungal memory Tc17 cells

One of the cardinal features of memory T-cell subsets is their ability to retain expression of signature cytokines. Effector Th17 cells are known to either cease IL-17A expression or convert into an IFNγ^+^ subset[17,23]. However, Th17/Tc17 cells are essential for immunity against many fungal and extracellular bacterial infections[37], and we have shown that loss of IL-17A signaling ablates vaccine-immunity[30,38]. Here, we investigated whether persistent, memory CD8^+^ eYFP^+^ T cells retain the ability to produce IL-17A. We found that ~30% (dLNs) to ~45% (spleen) of CD8^+^ eYFP^+^ cells expressed IL-17A (**Fig 1B**) following vaccination and, with a long period of rest after vaccination, the IL-17A expressing eYFP^+^ cells increased to 50%-60%, suggesting continuous maturation of memory Tc17 cells with loss of non-producers. We also observed similar frequencies of IL-17A^+^ cells among adoptively transferred CD8^+^ eYFP^+^ cells in WT and TCRα^-/-^ recipient mice (**Fig 1D**), suggesting that persistent vaccine antigen is dispensable for maintenance and fidelity under lymphopenic conditions. Thus, IL-17A expression in vaccine-induced memory Tc17 cells not only endured, but also increased during the memory phase.

### Antifungal memory Tc17 cells do not show plasticity towards IFNγ^+^ cells

In many studies involving infection, tumor, and autoimmunity, Th17 cells are either short-lived or convert into IFNγ producing cells[23]. Given the requirement that we previously observed for effector Tc17 cells following vaccination[30], we investigated whether antifungal memory Tc17 cells portray such plasticity. We detected significant numbers of eYFP^+^ IFNγ^+^ cells immediately after vaccination, especially in the spleen (**Fig 1B),** most of which did not produce IL-17A. However, the percentage of eYFP^+^ cells expressing IFNγ actually diminished over time. By day 346 post-vaccination <1% of eYFP^+^ cells in the dLN and ~3% of eYFP^+^ cells in the spleen were IFNγ^+^ (**Fig 1B**). Similar results were found in adoptively transferred CD8^+^ eYFP^+^ T cells, with <10% of them producing IFNγ in both WT and TCRα^-/-^ mice (**Fig 1D**). Next, we evaluated the effect of IL-12 and/or IFNγ on plasticity of memory Tc17 cells in an *in vitro* experiment. As expected, IL-12 stimulation significantly enhanced the frequency of IFNγ^+^ cells among eYFP^-^ CD44^hi^ (Tc1) cells (**S2A Fig**). However, memory Tc17 cells were resistant to IL-12- or IFNγ-induced signals for plasticity towards IFNγ production (**S2B Fig**) Thus, our data suggest that vaccine-induced anti-fungal *memory* Tc17 cells do not display plasticity towards IFNγ, even in the absence of persistent vaccine antigen or in a lymphopenic environment.

### Functional memory Tc17 cells for anti-fungal immunity

**Fig 2:**
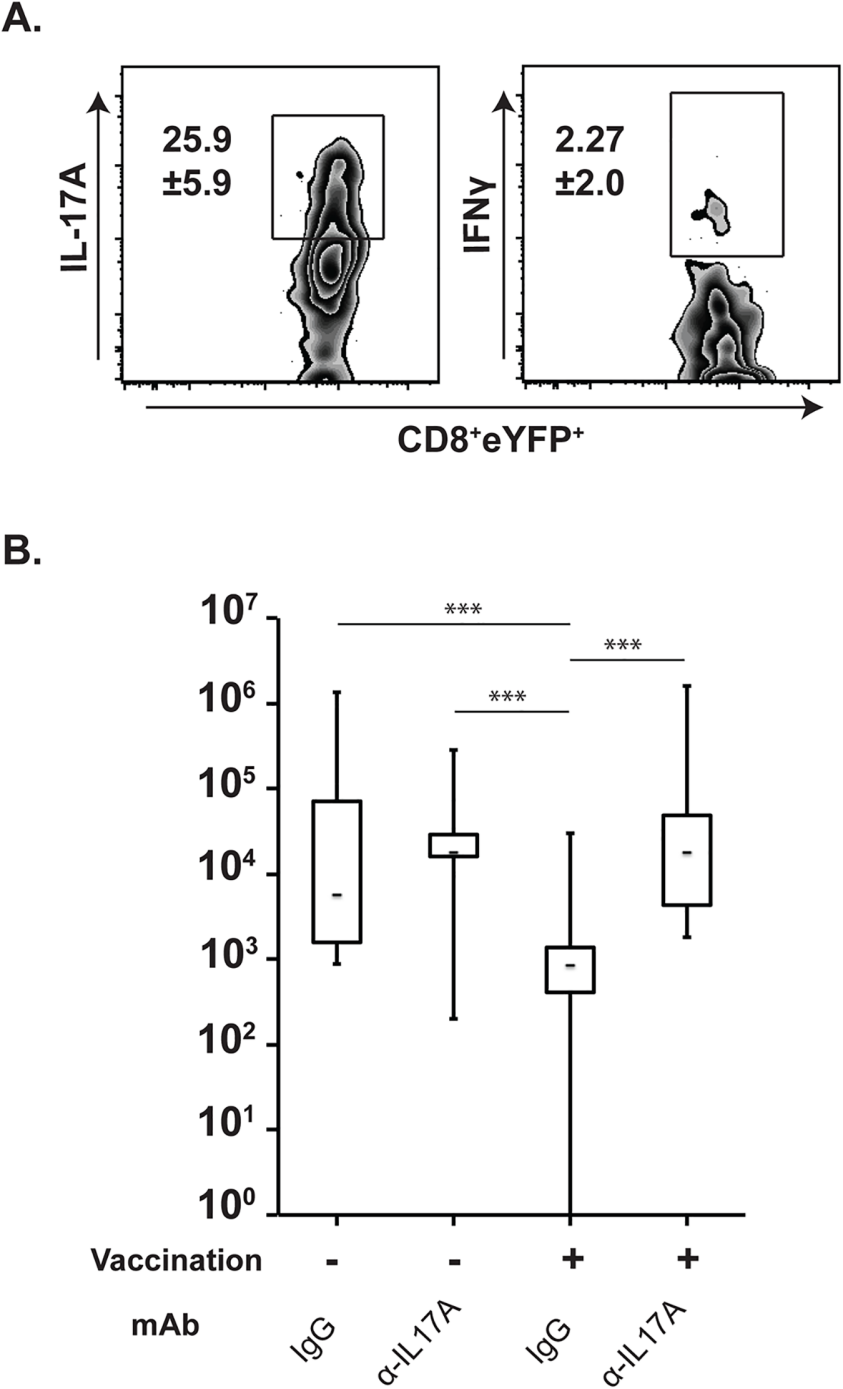
Functional role of memory Tc17 cells in resistance to fungal pneumonia. Recall: (**A**) Effector CD8^+^ T cells from vaccinated IL-17a^Cre^R26R^eYFP^ were purified from the dLNs and spleen on day 16 post vaccination and adoptively transferred into naïve WT recipients. After 5 months of rest, mice were challenged intratracheally with an isogenic virulent strain and after 4 days, lungs were harvested to analyze recall responses by flow cytometry. Values denote percent ± SD. Data is representative of 2 independent experiments. N = 4mice/group. Resistance: (**B**) Naïve WT mice were vaccinated with attenuated *B*. *dermatitidis* #55 strain and rested for ~5 months. Vaccinated mice and unvaccinated controls were challenged intratracheally with a virulent strain of *B*. *dermatitidis* (10^4^ CFU of #26199). Anti-IL-17A or control IgG antibody (300 μg) was administered intravenously every other day starting from day -1 of challenge. Lung homogenate was enumerated for CFU. Whisker plots represent data from two independent experiments harvested on day 6 and day 8 post-challenge. N = 17–23 mice/group. ***P≤0.001. CD4^+^ T cells were depleted throughout the entire 5-month experiment.

We and others have shown that *effector* Tc17 cells contribute to fungal resistance in the absence of CD4^+^ T cells[18,30,39]. Previously, we showed that *effector* Tc17 cells are obligatory for anti-fungal immunity, whereas type I cytokines (IFNγ, TNFα and GM-CSF) can be compensated[30,40]. Here, we investigated whether vaccine-induced *memory* Tc17 cells retain their recall responses and function in antifungal resistance over an extended period after vaccination. To test this issue, mice were rested for ~5 months after vaccination before we challenged them with a lethal strain of yeast. Upon pulmonary challenge, *memory* Tc17 cells efficiently recalled into the lung and expressed IL-17A (**Fig 2A**). However, *memory* eYFP^+^ Tc17 cells did not express IFNγ, suggesting a stable Tc17 lineage commitment without plasticity. To analyze the role of IL-17A in resistance, we neutralized soluble IL-17A with anti-IL-17A mAb throughout the infection after challenge. Unvaccinated mice had a higher fungal burden than vaccinated controls. Among vaccinated mice, the IL-17A neutralized group had a ~100-fold higher fungal burden than the mice treated with control antibody (**Fig 2**; P≤0.001), suggesting that memory Tc17 cells contributed significantly to vaccine-immunity in the absence of CD4^+^ T cells. Thus, vaccine-induced antifungal memory Tc17 cells persist and maintain the capacity to mediate protective immunity.

### Antifungal memory Tc17 cells express type I cytokines other than IFNγ

**Fig 3:**
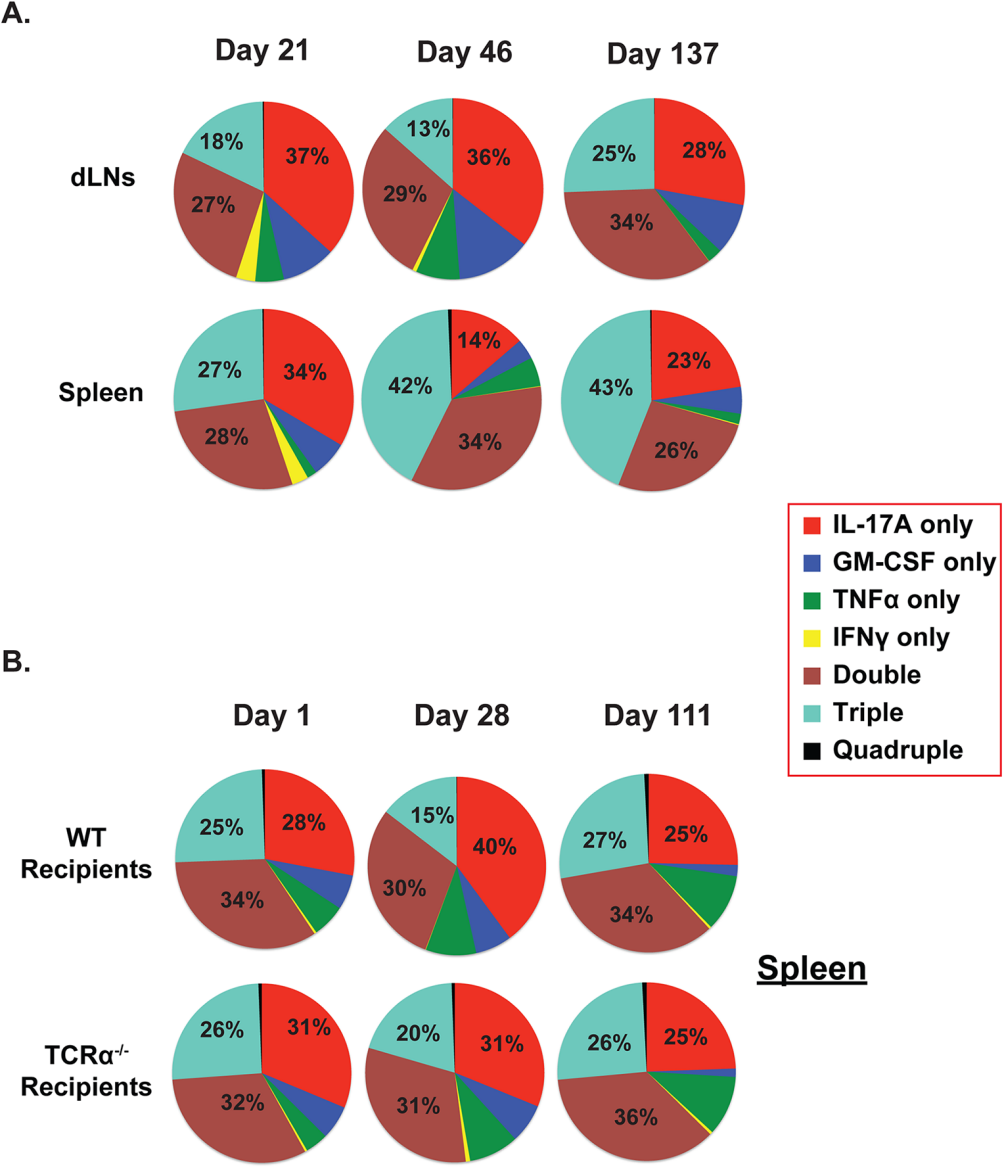
Multicytokine producing CD8^+^ eYFP^+^ T cells. Cells from mice portrayed in **Fig 1** were analyzed for expression of IL-17A, IFNγ, TNFα and GM-CSF cytokines. **(A)**. Naïve IL17a^Cre^R26R^eYFP^ mice were vaccinated with #55 strain. The draining lymph nodes (dLNs) and spleens were harvested on indicated days enumerate multi-cytokine producing CD8^+^ cells by flow cytometry. **(B)**. Naïve IL17a^Cre^R26R^eYFP^ mice were vaccinated, effector CD8^+^ T cells were purified (as described in Fig 1) and transferred into naïve WT or TCRα^-/-^ mice. On indicated days, cells were analyzed for multicytokine producing CD8^+^ T cells by flow cytometry. Values in the pie diagrams represent percent cytokine-expressing cells among all cytokine-producing cells.

We next asked whether CD8^+^ eYFP^+^ cells can produce cytokines other than IFN*γ*. Previously, we showed that type I cytokines such as GM-CSF and TNFα augment antifungal vaccine immunity[40]. Here, using cells portrayed in Fig 1, we assessed multi-cytokine production by *memory* CD8^+^ eYFP^+^ cells by analyzing the percentage of mutually exclusive single, double, triple and quadruple cytokine producing CD8^+^ eYFP^+^ cells (e.g. IL-17A, IFNγ, GM-CSF, and TNFα) (**Fig 3)**. *Effector* eYFP^+^ cells were largely single IL-17A producers, while most *memory* IL-17A^+^ eYFP^+^ cells expressed more than one cytokine with the largest pool in the spleen being triple producers by 137 days post vaccination (**Fig 3A)**. Notably, single or multicytokine IFNγ producing eYFP^+^ cells were lost during transition to the memory phase. Adoptively transferred effector eYFP^+^ cells were more often double producers than triple producers (**Fig 3B**), suggesting that prolonged antigen exposure may bias towards multi-cytokine producing memory Tc17 cells (**Fig 3A**). About 30% of eYFP^+^ cells produced none of these cytokines. We asked whether these cells had higher PD-1 expression, an indicator of a dysfunctional or exhausted phenotype. Only 12% of non-producers were PD-1^+^ (**S3A Fig**) similar to the proportion of producers expressing PD-1. We also looked at other T cell phenotypes: ≤5% of CD8^+^eYFP^+^ cells produced IL-22, a cytokine often associated with Th17 responses, and ≤1% expressed FoxP3^+^, which is associated with regulatory T cells (**S3B Fig**). We also evaluated whether memory IL-17 producing CD8^+^ T cells, similar to Th17 cells, co-express IL-21[41] along with IL-1R, IL-23R and Stat3. We found that Tc17 cells did not express IL-21, but they did express higher levels of IL-1R, IL-23R and Stat3 compared to Tc1 cells (**S3C Fig**) suggesting a distinct differentiation program in contrast to Th17 cells. Thus, vaccine induced *memory* Tc17 cells have the ability to produce multiple type I cytokines that confer fungal immunity, yet without undergoing conversion towards IFNγ production.

### Antifungal memory Tc17 cells are canonical CD8^+^ T cells with distinct phenotypic attributes

**Fig 4:**
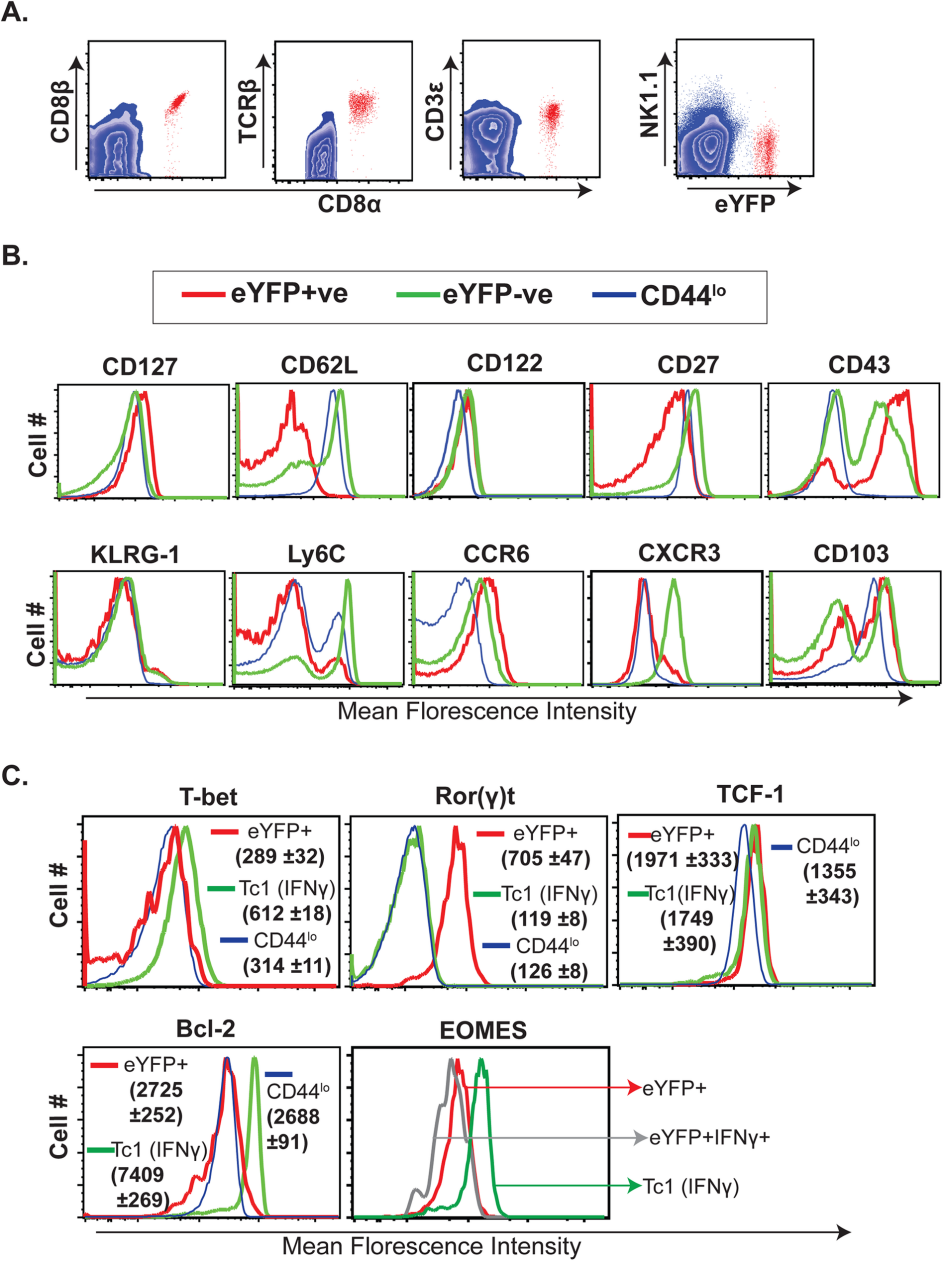
Phenotype and transcription factor profile of CD8^+^eYFP^+^ T cells. Splenocytes from vaccinated IL17a^Cre^R26R^eYFP^ mice (day 46 post-vaccination) were surface stained *ex vivo* for **(A)** CD8β, TCRβ, CD3ε, and NK1.1 expression on CD8α^+^ eYFP^+^ cells and **(B)** cytokine, chemokine, adhesion, co-stimulatory and terminal differentiation markers/ receptors. **C.** Naïve IL17a^Cre^R26R^eYFP^ mice were depleted of CD4^+^ T cells and vaccinated with strain #55. On day 346 after vaccination, splenocytes were re-stimulated and stained for surface markers, Bcl-2, intracellular cytokines and transcription factors. CD8^+^ T cell populations were analyzed by flow cytometry. Values represent mean florescence intensity (MFI) ±SD.

We evaluated the lineage specificity of CD8^+^ eYFP^+^ cells by looking at expression of CD8β, TCRβ and CD3ε on CD8α^+^ eYFP^+^ cells. CD8α^+^ eYFP^+^ cells equivalently co-expressed a CD8β chain (**Fig 4A**), a cardinal feature of conventional CD8^+^ T cells. Similarly, CD8α^+^ eYFP^+^ cells were TCRβ^+^ and CD3ε^+^, suggesting that memory Tc17 cells are indeed classical CD8^+^ T cells.

We characterized memory Tc17 cells for their phenotypic attributes and contrasted them with Tc1 cells (CD8^+^eYFP^-^) (**Fig 4B & S4 Fig**). Tc17 (eYFP^+^) cells expressed high levels of CD127 (IL-7Rα, essential for memory homeostasis); however, they were CD62L^lo^ and Ly6C^lo^, suggesting they are “effector” memory not “central” memory cells[10,42]. As expected for effector memory cells, none of the antifungal memory cells expressed the terminal differentiation marker KLRG-1[36,43]. Further, *memory* Tc17 cells retained high expression levels of chemokine receptor CCR6 and costimulatory molecule, CD43, similar to *effector* Tc17 cells[30]. In concordance with other studies[13,44], many of the Tc17 cells were CD27^lo^ [13,15,30]. Interestingly, most of the activated (CD44^hi^) T cells (both eYFP^+^ and eYFP^-^) expressed integrin CD103 (αE), the alpha chain of integrin αE β7 that selectively marks tissue “resident” memory (T_RM_) cells[45].

We also compared the transcription factor profile of memory Tc17 cells with Tc1 cells (**Fig 4C**). Memory Tc17 cells retained high expression of the prototypical transcription factor Ror(γ)t, and expressed low levels of transcription factors T-bet and Eomes typically associated with Tc1 cells. Notably, the expression levels of TCF-1, a LEF/TCF family member associated with memory and stem-cell like activity[13,46], was similar or higher in *memory* Tc17 cells vs. *memory* Tc1 (CD44^hi^eYFP^-^) cells. Likewise, the frequencies of many phenotypic attributes of Tc17 cells differed significantly from eYFP^-^CD44^hi^ cells (Tc1) and naïve CD8^+^ T cells (**S4 Fig**). Collectively, our data suggest that memory Tc17 cells are canonical CD8^+^ T cells that display distinct phenotypic attributes and constitute an “effector memory” population.

### Basal homeostatic proliferation of memory Tc17 cells is higher than memory Tc1 cells

**Fig 5:**
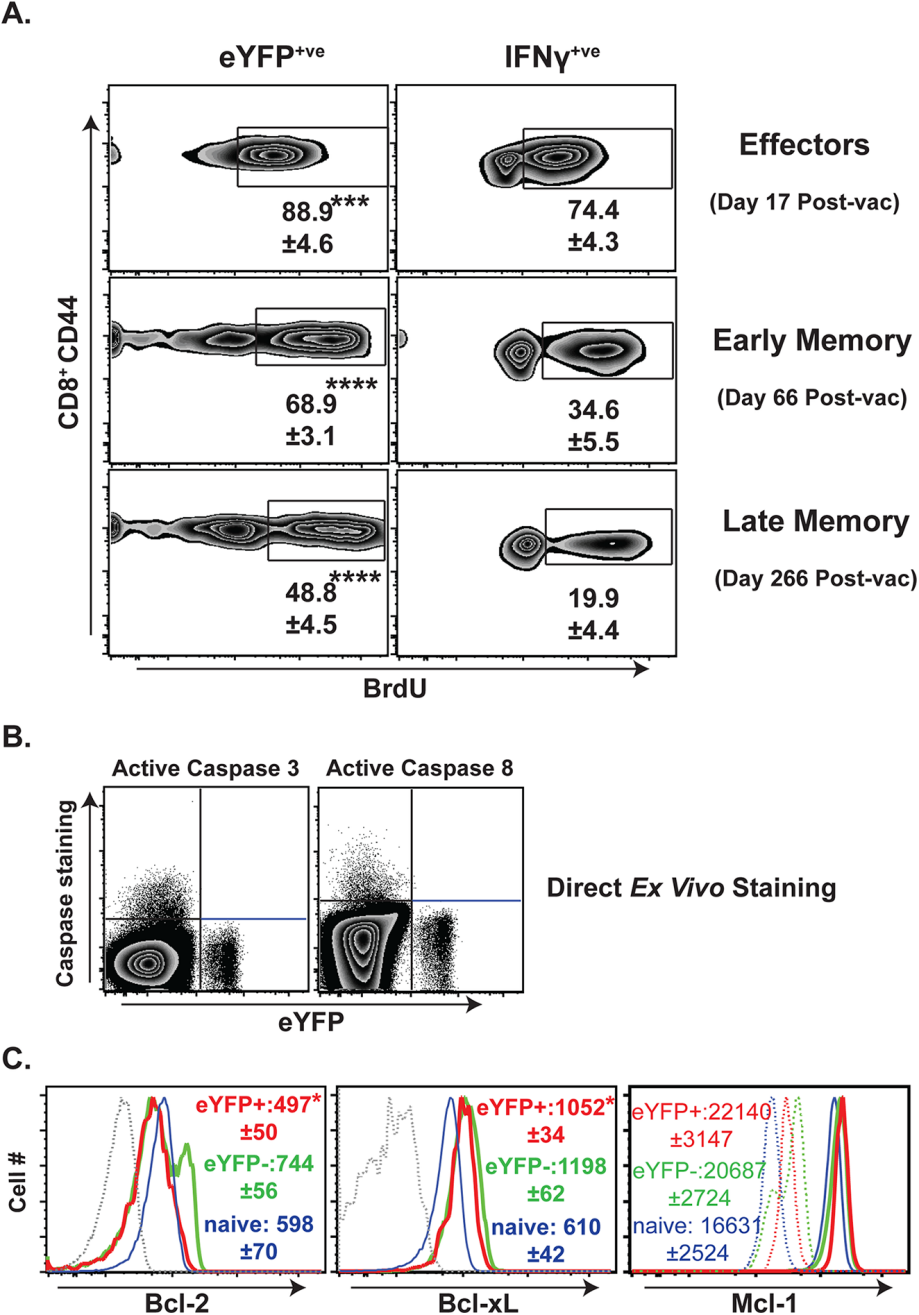
Proliferation and apoptosis profile of anti-fungal memory CD8^+^ T cells. Naïve IL17a^Cre^R26R^eYFP^ mice were depleted of CD4^+^ T cells and vaccinated as noted in Fig 1. **A.** Proliferation of anti-fungal effector cells, and early and late memory CD8^+^ T cells. Vaccinated mice were pulsed with BrdU in drinking water (DW) for 12 days. On the following day, spleens were harvested, processed and re-stimulated. Cells were surface-stained and stained intracellularly for cytokines before analysis of BrdU staining. Numbers represent the percent BrdU^+^ of eYFP^+^/IFNγ^+^ CD8^+^ T cells. N = 5 mice/group. **B.** On day 76 post-vaccination, surface-stained splenocytes were stained intracellularly for active Caspase3/8. Dot plots show the staining of pro-apoptotic caspases gated on CD8^+^CD44^hi^ T cells. N = 4–5 mice. **C.** On day 76 post-vaccination, splenocytes were stained for surface markers before staining for intracellular anti-apoptotic factors. Values indicate MFIs gated on CD8^+^CD44^hi^ eYFP^+/-^ T cells. Dotted lines indicate staining controls. Data are representative of two independent experiments. *P≤0.05, ***P≤0.001, and ****P≤0.0001.

Th17 cells generally display more proliferative renewal than Th1 cells, but homeostatic renewal of Tc17 cells is less understood[47]. We have shown that antifungal *effector* Tc17 cells undergo significantly higher proliferation than *effector* Tc1 cells during the expansion phase after vaccination[39]. Here, we assessed the proliferative ability of early and late *memory* Tc17 and Tc1 cells (**Fig 5A & S5 Fig**). Consistent with other studies[48], *memory* Tc1 cells became quiescent, and the proliferation of *memory* Tc1 cells (≈20%) was ~3.7 times lower than *effector* Tc1 cells (≈75%) as measured by BrdU uptake over a 12-day pulse. Although *memory* Tc17 cells also showed decreased basal homeostatic proliferation compared to *effector* Tc17 cells (~1.8-fold reduction), proliferation remained significantly higher in *memory* Tc17 cells than *memory* Tc1 cells (49% vs. 20%). Thus, vaccine-induced *memory* Tc17 cells appear to have a higher proliferative renewal than *memory* Tc1 cells.

Homeostatic turnover of CD8^+^ T cells involves a similar number of cells undergoing apoptosis in order to maintain constant numbers[48]. Based on our BrdU data, we expected that *memory* Tc17 cells would display higher levels of apoptosis than Tc1 cells. Surprisingly, eYFP^+^ cells did not display staining for active caspase3/8 (**Fig 5B**), albeit the cells were tested at a single time-point (76 days after vaccination). Likewise re-stimulation with anti-CD3 and -CD28 to enhance apoptosis revealed that *memory* Tc17 cells were indeed resistant to TCR signal-induced apoptosis (**S6 Fig**). We also measured the levels of anti-apoptotic factors (e.g. Bcl-2, Bcl-xL, Mcl-1) and observed that *memory* Tc17 cells expressed significantly lower levels of Bcl-2 and Bcl-xL and similar levels of Mcl-1 compared with *memory* Tc1 cells (**Fig 5C**). *Memory* Tc17 cells tended to express greater levels of these anti-apoptotic factors than naïve CD8^+^ T cells. Thus, antifungal *memory* Tc17 cells are maintained with higher levels of proliferation renewal than Tc1 cells, and although memory Tc17 cells express lower levels of the anti-apoptotic factors Bcl-2 and Bcl-xL, they have greater resistance to apoptosis.

### Memory Tc17 cell homeostasis is independent of anti-apoptotic factor Bcl-2

**Fig 6:**
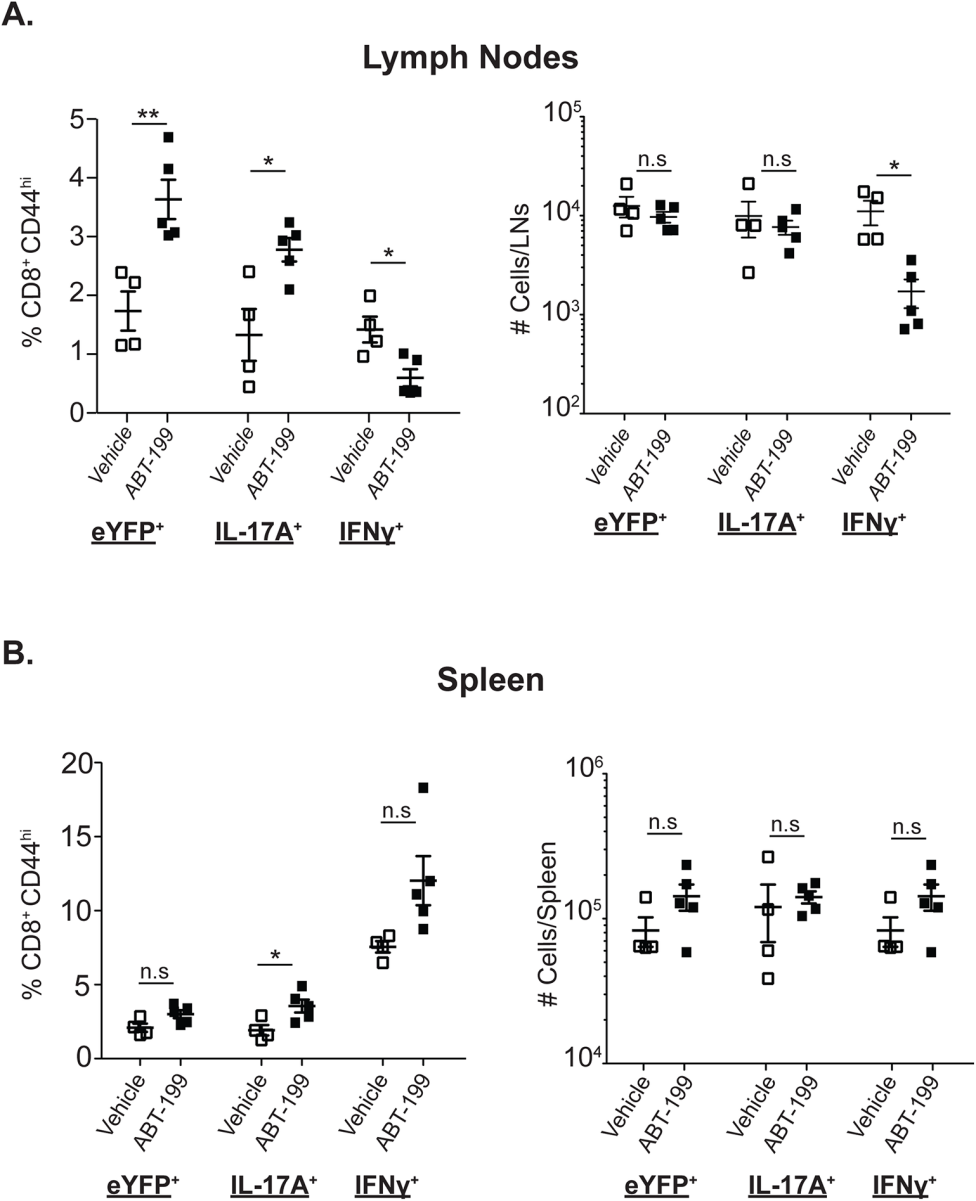
Role of Bcl-2 for memory Tc17 cells. IL17a^Cre^R26R^eYFP^ mice were vaccinated and rested for ~90 days. Mice were treated with either vehicle or Bcl-2 inhibitor ABT-199 (20 mg/kg body weight) for 10 days. Lymph nodes (**A**) and spleens (**B**) were harvested to analyze memory CD8^+^ T cells positive for eYFP, IL-17A and IFNγ. Data is representative of 4–5 mice/group. CD4^+^ T cells were depleted throughout the experiment. *P≤0.05 and **P≤0.01.

Memory Th17 cells express higher levels of Bcl-2 than memory Th1 cells[12,13], linked to resistance to cell death[12]. However, our data revealed that anti-fungal memory Tc17 cells expressed lower levels of Bcl-2 than memory Tc1 cells; the Bcl-2 expression levels in memory Tc17 cells were nevertheless on par with naïve T cells (**Figs 4C & 5C**), which require Bcl-2 for survival[49]. To investigate the role of Bcl-2 for memory Tc17 cell homeostasis *in vivo*, we inhibited Bcl-2 chemically with ABT-199 in vaccinated mice and analyzed memory CD8^+^ T cells. The total numbers of CD8^+^ T cells, both activated and naïve, were significantly reduced in lymph nodes but not spleens (**S7A Fig),** indicating that Bcl-2 inhibition reduced lymph node size. Bcl-2 inhibition also significantly decreased the frequency and total numbers of IFNγ^+^ (Tc1) cells in the lymph nodes (**Fig 6A**), but not in the spleens. Central memory T cells are enriched in the lymph nodes suggesting that ABT-199 preferentially affects survival of central memory IFNγ^+^ (Tc1) cells as previously described[50]. Despite the significant reduction in the total CD8 T-cell population and memory Tc1 cells in the lymph nodes upon Bcl-2 inhibition (**S7A Fig**), the total number of IL-17A^+^ CD8 T-cells was unchanged in the draining lymph nodes (**Fig 6A**). The frequencies and total numbers of CD8^+^eYFP^+^ (Tc17) cells were unaffected in the spleens, as with Tc1 cells. Although Tc1 cells in the lymph nodes responded to Bcl-2 inhibition with increased proliferation, as assessed by Ki67 staining, Tc17 cells did not (**S7B Fig**). Collectively, these results suggest that Bcl-2 is required for the maintenance and survival of anti-fungal central memory Tc1 cells, but not Tc17 cells.

### HIF-1α is required for differentiation but not for sustenance of effector Tc17 cell response

**Fig 7:**
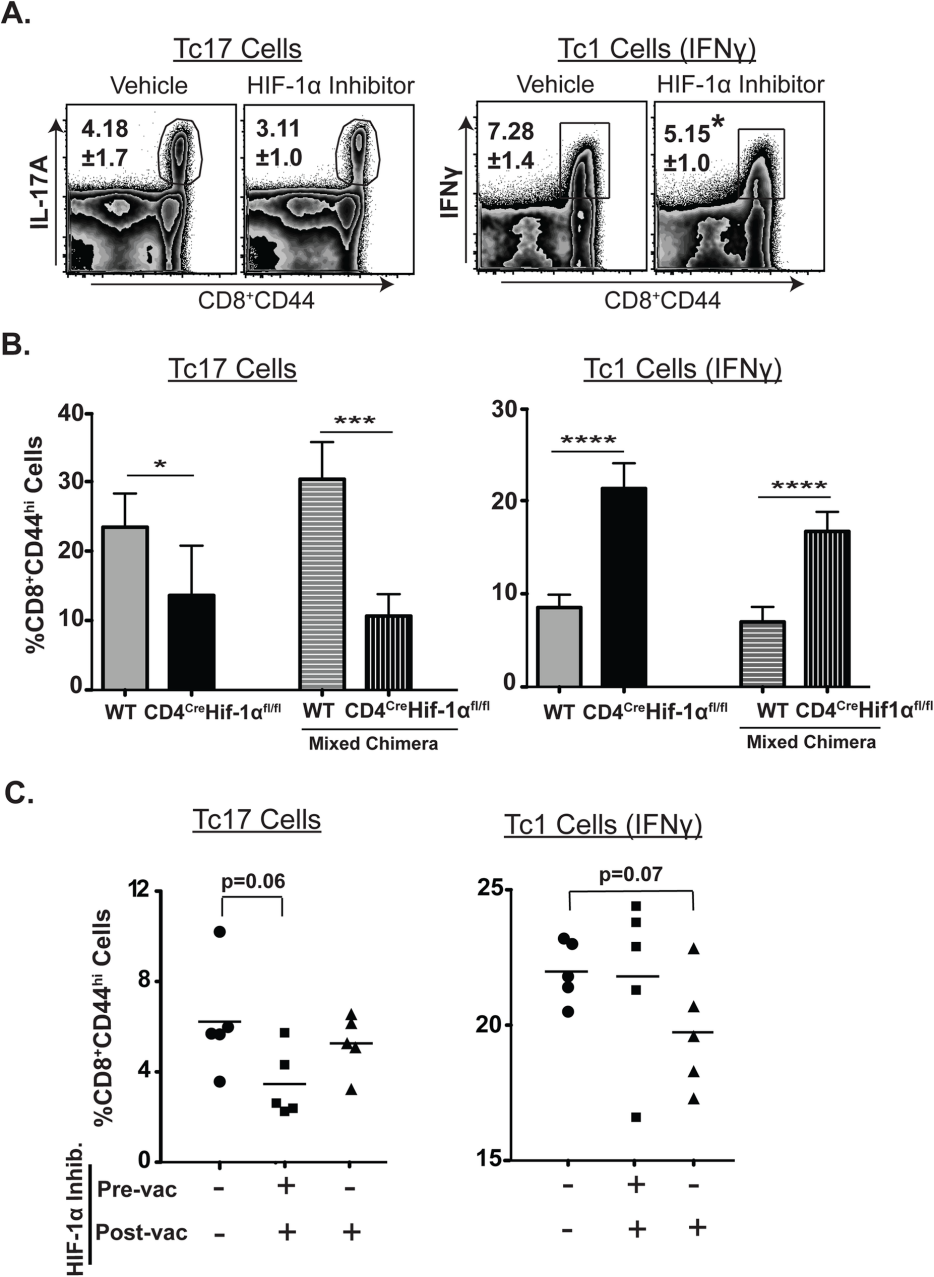
Impact of HIF-1α on proliferation of effector Tc17 and Tc1 cells. Naïve IL17a^Cre^R26R^eYFP^ or chimera mice were depleted of CD4^+^ T cells and vaccinated with strain #55. Splenocytes were re-stimulated, surface-stained, and stained for intracellular cytokines. (A) Mice were given Echinomycin intraperitoneally every other day (starting day 4 post-vaccination) for 10 days to inhibit HIF-1α. Percent cytokine-producing cells among polyclonal CD8^+^ T cells (day 15 post-vac). **B.** Percent cytokine producing cells gated on CD8^+^CD44^hi^ T cells in single and mixed bone marrow chimera mice (day 17 post-vac). **C**. Mice were given Echinomycin every other day starting at -7 days (pre-vac) or at +4 days (post-vac) of vaccination. Percent cytokine producing cells gated on CD8^+^CD44^hi^ T cells (day 17 post-vac). N≥5 mice/group. Data is representative of at least five (**A**) and two (**C**) independent experiments. Values are mean ± sd. *P≤0.05, ***P = 0.001, ****P≤0.0001.

Hypoxia-inducible factor 1α (HIF-1α) plays a role during the induction and survival of Th17 cells[12,51]. HIF-1α also regulates the expression of Bcl-2 indirectly through Notch signaling in Th17 cells[12]. To our knowledge, the role of HIF-1α for generation of antifungal *effector* Tc17 (or Tc1) cells has not been investigated. We first investigated the expression levels of HIF-1α in CD8^+^ eYFP^+^ vs. eYFP^-^ cells. Basal expression of HIF-1α in both effector and memory CD8^+^ eYFP^+^ cells was low, but increased upon re-stimulation (**S8A & S8B Fig**). We next investigated the functional role of HIF-1α on Tc17 and Tc1 cells by using the chemical inhibitor Echinomycin to block HIF-1α during the expansion phase, begun 4 days after vaccination. The proportion of *effector* Tc1 cells was significantly reduced by inhibition of HIF-1α, whereas the proportion of *effector* Tc17 cells was unaffected, indicating that HIF-1α is required for *effector* Tc1 cells but not for the sustenance or function of effector Tc17 cells (**Fig 7A**). In a complementary approach, we generated bone-marrow chimera mice using CD4^cre^HIF-1α^fl/fl^ bone marrow cells. Following vaccination, we harvested spleens to assess the expansion of Tc17 and Tc1 cells. In contrast to Echinomycin treatment, we found a significant reduction in Tc17 cells that lacked HIF-1α intrinsically and a significantly augmented Tc1 response (**Fig 7B**). To reconcile discrepant findings (**Fig 7A & 7B**), we began Echinomycin treatment before vaccination and continued it afterward to mimic HIF-1α deficient chimeric mice. We found that Tc17 responses were reduced when HIF-1α was blocked both before and after the vaccination, in contrast to results when HIF-1α was blocked only after vaccination (**Fig 7C**). These results reconcile the disparate findings of HIF-1α inhibitor treatment and HIF-1α^-/-^ chimeric mice (**Fig 7A & 7B**) and suggest HIF-1α is required for differentiation but not sustenance of effector Tc17 cell responses.

### Temporal blockade of HIF-1α shrinks memory Tc17 cells

**Fig 8:**
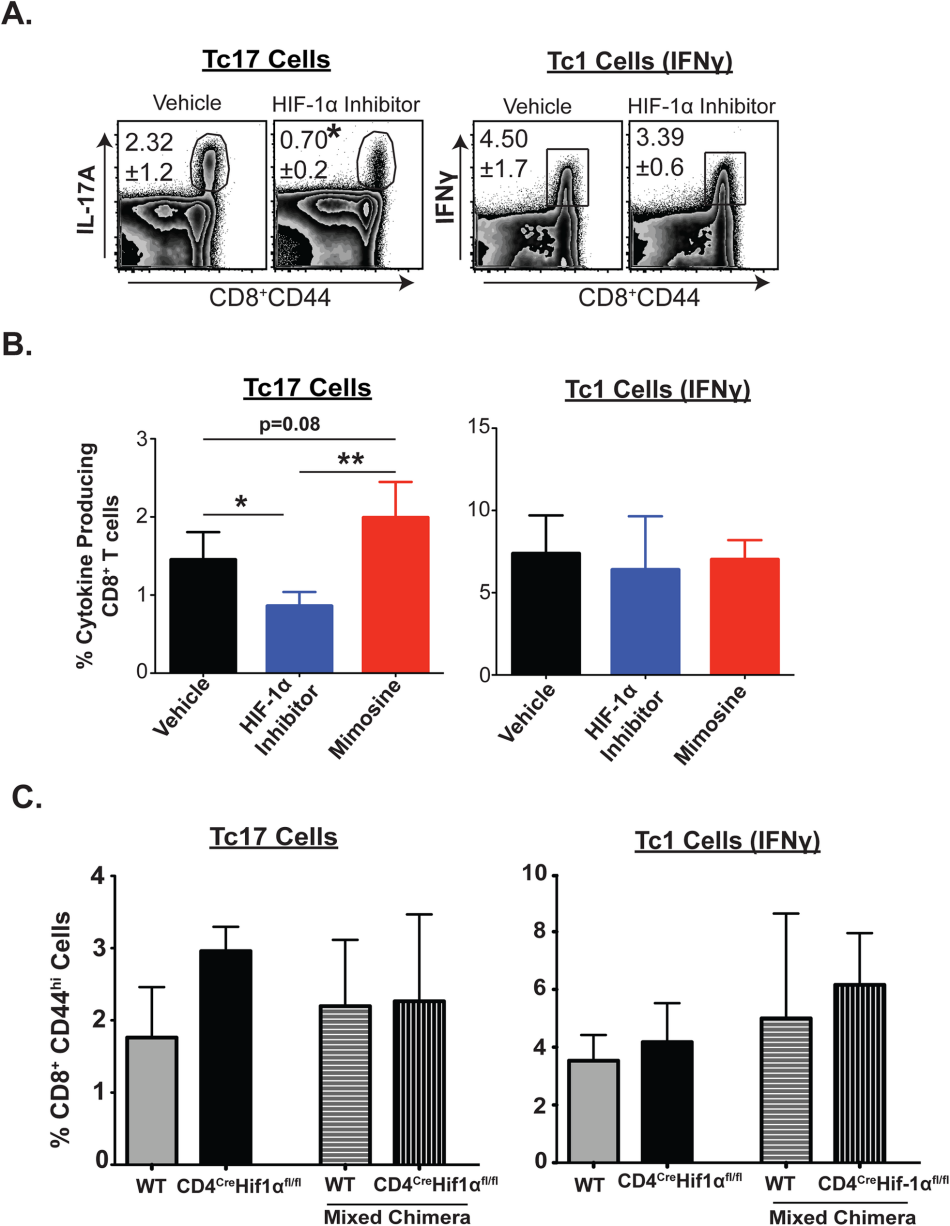
Impact of HIF-1α on homeostasis of memory Tc17 and Tc1 cells. Naïve IL17a^Cre^R26R^eYFP^ or chimeric mice were depleted of CD4^+^ T cells and vaccinated with strain #55. Mice were rested for at least 90 days. **A.** Echinomycin was administered for 10 days beginning 90 days after vaccination. On day 11, spleens were harvested to analyze cytokine producing CD8^+^ T cells. **B.** Mice were given Echinomycin or Mimosine every other day as above for 14 days during the memory phase, Percent cytokine-producing cells among polyclonal CD8^+^ T cells (**A & B**) **C.** Percent cytokine producing cells gated on CD8^+^CD44^hi^ T cells in single and mixed bone marrow chimera mice. Data represents percent cytokine-producing cells among CD8^+^ T cells in spleens. Values are mean ± SD. N≥5 mice/group. Data is representative of at least five (**A**) and two (**C**) independent experiments. *P≤0.05.

We showed above that differentiation but not sustenance of effector Tc17 cell responses requires HIF-1α. To our knowledge, the role of HIF-1α during homeostasis of *memory* Tc17 (or Tc1) cells has not been investigated. We asked here whether HIF-1α is required for memory homeostasis of Tc17 and Tc1 cells. We took two pharmacological approaches. First, we gave Echinomycin for 10 days at day 90 after vaccination to temporally block HIF-1α activity (**Fig 8A**). The proportion of *memory* Tc17 cells was significantly reduced by inhibitor treatment, whereas the proportion expressing IFNγ was not affected, indicating that HIF-1α is required for IL-17A expressing memory cells. We further analyzed the requirement for HIF-1α in *memory* Tc17 cells by gating on CD8^+^ eYFP^+^ cells (**S8D Fig**). The frequency of memory CD8^+^eYFP^+^ cells was similar for the vehicle and inhibitor-treated groups (**S8C Fig**), suggesting that HIF-1α chiefly affects cytokine expression. In a second pharmacological approach, we administered the HIF-1α agonist, Mimosine[52], for 14 days starting at day 90 after vaccination (**Fig 8B**). In contrast to the HIF-1α inhibitor, the agonist enhanced memory Tc17 cells, but again did not affect memory Tc1 cells, buttressing evidence for the selective requirement of HIF-1α for memory Tc17 homeostasis.

Above, using chimeric mice that lack HIF-1α (**Fig 7B**), we observed that T cell intrinsic HIF-1α is required for the differentiation of Tc17 cells. To assess the fate of effector Tc17 cells generated under these conditions, we rested these vaccinated mice for 90 days before analysis of Tc17 memory. Memory Tc17 (and Tc1 cells) were unexpectedly similar in all groups (**Fig 8C**), suggesting a limited intrinsic role for HIF-1α during memory CD8^+^ T-cell homeostasis generated in the absence of HIF-1α. Our data together argue that while temporal blockade of HIF-1α negatively impacts memory Tc17 cell homeostasis, some memory Tc17 cells may be generated and persist independent of intrinsic HIF-1α or alternatively extrinsic sources of this factor may compensate for loss of intrinsic HIF-1α.

## Discussion

The long term persistence and plasticity of Th17 cells varies in different experimental models. The importance of these features carries particular significance in the setting of vaccine immunity where maintenance and fidelity of the phenotype is often required for durable resistance to infection. Here, we studied vaccine-induced Tc17 cells that are essential for resistance against lethal fungal pneumonia in hosts that lack CD4^+^ T cells. We report that vaccine-induced antifungal *memory* Tc17 cells are highly durable over a prolonged period of nearly one year in a murine model. The cells stably persist as memory cells, retain the ability to express IL-17A, mediate immunity upon challenge, do not undergo plasticity towards IFNγ (yet do also express other type I cytokines important for fungal immunity), undergo high proliferative renewal, portray phenotypic markers that are consistent with “effector memory” cells, and are dependent on functional HIF-1α for homeostasis but not on Bcl-2 for survival.

Whereas Th17 cells induced by systemic or mucosal bacterial infection are short-lived memory cells characterized by loss of CD27 expression[15], we observed long-term persistence of antifungal Tc17 memory cells despite their low expression of CD27. CD27 is a costimulatory molecule known to augment Tc1 cell proliferation and function, but it may be nonessential for Tc17 cell functions and compensated by other co-stimulatory molecules/cytokines such as CD43, TLR2, IL-1β, and IL-23[11,30,53,54]. Previous studies have reported the persistence of CD27^lo^ Th17 cells following their transfer into animals after *in vitro* polarization[12,13,17]. The anti-fungal memory Tc17 cells investigated herein expressed high levels of TCF-1, which is a marker of a stem cell-like signature[13]. It is not clear if mucosal-induced Th17 cells can become *in situ* memory cells in the absence of cognate antigen. Nevertheless, several *in vivo* and *ex vivo* studies in mice and humans have shown that Th17 cells can become long-term memory cells[12,13,47]. Taken together, several lines of evidence argue that Th17 cells are able to persist despite their low expression of CD27. We extend these observations to include Tc17 cells.

Studies of antifungal T cell responses have yielded conflicting results concerning persistence and fidelity of Th17 cells. A murine model of oropharyngeal (mucosal) *Candida* infection revealed that Th17 cells persist for weeks[18]. In contrast, intradermal acute *Candida* infection in mice induced Th17 cells that quickly lost IL-17A production[17]. Herein, we used a vaccination model in mice to track the persistence and fidelity of Tc17 cells for nearly one year. We observed that Tc17 cells were robustly induced and they durably maintained their IL-17-producing phenotype for the entire period. Th17 cells induced during a cutaneous *Candida* infection might migrate to secondary lymphoid organs, but the transient exposure to antigen and unique microenvironment during acute cutaneous *Candida* infection may not be enough to stabilize a Th17 cell phenotype for memory T cell formation. Nevertheless, our adoptive transfer studies demonstrated that vaccine-induced CD8^+^ eYFP^+^ T cells persist and produce IL-17A even in the absence of vaccine antigen.

In many models, Th17 cells convert into IFNγ producing cells[23]. In the current study, however, we observed little conversion of Tc17 cells into IFNγ producing Tc1 cells, although the cells expressed TNFα and GM-CSF (**Fig 3**). The vaccine-induced effector CD8^+^ eYFP^+^ donor cells that were adoptively transferred produced comparable levels of IL-17A in both WT and immunodeficient TCRα^-/-^ recipients, and little plasticity towards IFNγ, implying the importance of a distinct initial microenvironment for phenotype stabilization[24,55]. Interestingly, the percentage of antifungal Tc17 cells producing multiple cytokines increased when the cells were exposed to vaccine antigen for an extended period (**Fig 3**). One possible explanation is that prolonged exposure to fungal ligands and an inflammatory milieu may stabilize the polycytokine-producing phenotype of Tc17 cells[56]. Further studies are needed to elucidate how and what microenvironment shapes the stable Tc17 responses following fungal vaccination.

Memory Th1/Tc1 cells are subdivided into central or effector memory cells distinguished by the surface markers CD62L and CCR7, respectively[10]. Our data showed that antifungal Tc17 cells are effector memory cells as observed in human memory Th17 cells[12]. Notably, antifungal memory Tc17 cells retained expression of the prototypical transcription factor Ror(γ)t, while expressing lower levels of T-bet. Our data also showed that the majority of memory Tc17 cells expressed surface CD103, an integrin marker of tissue resident memory cells, although the significance of this marker for persistent antifungal immunity needs further study.

In general, homeostasis of memory T cells is governed by two key cytokines, IL-7 and IL-15, which are part of the common γ_c_ cytokine family. We found that *memory* Tc17 cells display high levels of CD127 (IL-7Rα chain) and CD122 (IL-2Rβ chain), implying similarity with memory Tc1 cell homeostasis. Nevertheless, we found that *memory* Tc17 cells belong to the class of “effector memory”, and in contrast to effector memory Tc1 cells, Tc17 cells undergo higher proliferation renewal[5]. The cytokines, IL-7 and IL-15, are required for intermittent proliferation and also promote survival by enhancing anti-apoptotic factors such as Bcl-2 and Bcl-xL, and downregulating apoptotic factors[57,58]. Our studies using a specific Bcl-2 inhibitor revealed that Tc17 cells were unaffected, while central memory Tc1 cells were reduced in the draining lymph nodes and accompanied by increased cell proliferation. Our findings suggest that memory Tc17 cell homeostatic survival is independent of Bcl-2 and that Tc17 cells may depend on the other anti-apoptotic factors for their survival. While central memory Tc1 cells require Bcl-2 [50], the low levels of Bcl-2 in Tc17 cells probably makes them resistant to Bcl-2 inhibition and may promote their high basal proliferative renewal.

HIF-1 family members are transcription factors that regulate survival and function of many cells, including T cells under hypoxic conditions, while also promoting carcinogenesis[59]. Abrogation of HIF-1α inhibits differentiation of Th17 cells, and blockade leads to apoptosis of Th17 cells[12,51]. Ablation of the HIF-1 negative regulator, VHL, boosts cytolytic CD8^+^ T cell responses, enhancing viral clearance and suppressing tumor growth. Here, we found disparate requirements for HIF-1α during effector and memory Tc17 responses and also between Tc17 and Tc1 cells. After vaccination, during the expansion phase, antifungal Tc1 (IFNγ^+^) cells required HIF-1α for their sustenance of effector cell responses, a feature consistent with prior work on Tc1 cells exposed to persistent antigen[60]. Antifungal Tc17 cells were different: while differentiation of *effector* Tc17 cells required HIF-1α, in line with Th17 cells in a prior study[51], sustenance of *effector* Tc17 during the expansion phase did not required HIF-1α. Of note, genetic ablation of HIF-1α intrinsically in T cells or extended use of an HIF-1α inhibitor begun prior to vaccination enhanced the Tc1 cell responses. This is likely due to a reciprocal increase of Tc1 cells over Tc17 cells[61], partly caused by poor induction of RORγt in HIF-1α^-/-^ T cells[62].

*Memory* Tc17 cells required HIF-1α for their homeostasis and maintenance of an IL-17A expression phenotype, and this requirement distinguished memory Tc17 and Tc1 cells. This assertion is supported by the results of temporal intervention with Echinomycin in vaccinated mice. Conversely, our bone-marrow chimera studies revealed that a population of effector Tc17 cells lacking HIF-1α^-/-^ intrinsically became memory cells without loss of phenotype or numbers, suggesting either HIF-1α independent generation of memory Tc17 cells or compensation from the extrinsic compartment. We do not know the oxygen level in the microenvironment of antigen presentation and CD8^+^ T cell expansion *in vivo*; however, *effector* Tc17 cells may overcome the requirement of HIF1-α following differentiation while *memory* Tc17 cells may have a distinct immunometabolism[63,64], accounting for the HIF-1α requirement for maintenance of an active IL-17A locus in addition to survival during the memory phase. Further studies using cell-specific inducible HIF-1α are required to dissect its role in effector and memory Tc17 homeostasis. It is notable that *memory* Tc1 cells were relatively unaffected by inhibition of HIF-1α, suggesting that anti-neoplastic drugs such as Echinomycin might be used without affecting some tumor-specific memory CD8^+^ cell functions.

In conclusion, we have shown that antifungal Tc17 cells display a set of appealing and unexpected features that are relevant to their function in a vaccine setting. These vaccine-induced Tc17 cells persist as long-lasting memory cells without undergoing plasticity towards IFNγ production, while they express other type I cytokines and have high levels of proliferative renewal for homeostasis. Tc17 cells expressed low levels of Bcl-2 compared to Tc1 cells, and Bcl-2 was not required for the maintenance of memory Tc17 cells. Memory Tc17 cells here were uniquely dependent on functional HIF-1α for the expression of the signature cytokine IL-17A. These results may inform the rational design of vaccines that are dependent on long-lived IL-17A producing cells and perhaps guide the development of immune modulators to alleviate immunopathology and autoimmunity, or enhance immunity against cancer.

## Methods

### Mice

Wild type C57BL/6 and B6.SJL-*Ptprc*^*a*^*Pepc*^*b*^/BoyCrl mice were obtained from National Cancer Institute/Charles River Laboratories. Breeding pairs of IL17a^tm1.1((icre)Stck^/J (Stock 016879) and B6.129X1-Gt(ROSA)26Sor^tm1(EYFP)Cos^/J (Stock 006148) were purchased from Jackson Laboratories and were intercrossed to maintain a heterozygous IL-17A^cre^ locus in order to analyze both eYFP and functional IL-17A protein expression[17]. Homozygous breeding pairs of B6.129S2-TCRa^tm1Mom^/J and B6.PL-*Thy1*^a^/CyJ also were purchased from Jackson Laboratories. Conditional HIF-1α KO (CD4^Cre^HIF-1α^fl/fl^) mice were a generous gift from Dr. Fan Pan (School of Medicine, John Hopkins University). All mice were 7–8 weeks of age at the time of vaccination. Mice were bred, housed and cared for following strict guidelines of the University of Wisconsin Animal Care Committee, who approved all aspects of this work.

### Ethics statement

All animal procedures were performed in accordance with the recommendations in the Guide for the Care and Use of Laboratory Animals of the National Institutes of Health. Care was taken to minimize animal suffering. The work was done with the approval of the IACUC of the University of Wisconsin-Madison who approved the relevant animal protocol number MOO969.

### Fungi, vaccination and infection

The wild-type virulent strain of *Blastomyces dermatitidis* was purchased from American Type Culture Collection (ATCC; strain #26199). The isogenic, attenuated mutant lacking BAD1 (strain #55) was used for all vaccinations[38]. Isolates of B. *dermatitidis* were cultured and maintained as yeast on Middlebrook 7H10 agar with oleic acid-albumin complex (Sigma-Aldrich) at 39°C. Mice were vaccinated with *B*. *dermatitidis strain* #55 (~5 x 10^5^cfu) subcutaneously at two sites, dorsally and at the base of tail. For challenge studies, the virulent *B*. *dermatitidis* strain #26199 (~10^4^ cfu) was inoculated intratracheally. To ennumerate fungal burden, lung tissue was homogenized and plated on brain heart infusion (BHI; Difco) agar.

### Antibodies

All antibodies were purchased from BD Bioscience except antibodies directed against CD43 (clone 1B11), KLRG-1 and CXCR3 obtained from Biolegend; CD44, CD127, IL-22, T-bet, Ror(γ)t, Eomes, IL-21, FoxP3 from eBioscience; Bcl-2, Bcl-xL, Mcl-1 active Caspase 3, active Caspase 8, TCF-1 from Cell Signaling; HIF-1α from Novus Biologics, and GFP from Life Technologies.

### CD4^+^ T-cell depletion and IL-17A neutralization

All experiments were done in mice depleted of CD4^+^ T-cells; GK1.5 mAb was given in a weekly dose of 100μg/mouse by the intravenous route. The dose and interval was enough to deplete CD4^+^ T cells with an efficiency of 95%[36]. For *in vivo* IL-17A neutralization, mice were given 300 μg of anti-IL-17A mAb intravenously every other day. Both mAbs were purchased from Bio X Cell, West Lebanon, NH, while control IgG for IL-17A neutralization studies was obtained from Sigma-Aldrich.

### Intracellular cytokine staining

Single cell suspensions were re-stimulated with anti-CD3 (0.1 μsg/ml) and anti-CD28 (1 μg/ml) antibodies in the presence of Golgi Stop at 37°C for 5 hrs. Cells were surface-stained in 2% BSA/PBS buffer, fixed with Fix/Perm buffer (BD Biosciences) and stained for intracellular cytokines in 1X Perm/Wash buffer (BD Biosciences). Cytokine-producing CD8^+^ T cells were analyzed by flow cytometry using BD LSRII and FACS Aria.

### Adoptive transfers

Single cell suspensions from lymph nodes and spleens were subjected to a CD8^+^ T-cell magnetic bead-enrichment kit (BD Bioscience) to purify CD8^+^ T cells. Numbers of CD8^+^ eYFP^+^ cells were enumerated by flow cytometry, and equal numbers of CD8^+^ eYFP^+^ cells were adoptively transferred into naïve WT and TCRα^-/-^ recipient mice by the intravenous route.

### Transcription factors staining

Single cell suspensions were re-stimulated, surface-stained and fixed with Phosflow Lyse/Fix buffer and Phosflow Perm/Wash buffer (BD Biosciences). Cells were then stained for intracellular cytokines and transcription factors (TCF-1, Ror(γ)t and T-bet) simultaneously. Staining of FoxP3, HIF-1α and EOMES was done using a FoxP3 buffer kit (eBioscience). In some experiments, cells were first fixed with Perm/Fix buffer (BD Bioscience) followed by addition of antibodies to stain cytokines and eYFP (anti-GFP antibody), then subjected to transcription factor staining.

### Apoptosis factor staining

Cells were first surface-stained followed by intracellular staining for apoptosis factors using the BD Perm/Fix buffer kit. In some experiments, cells were subjected to intracellular staining for cytokines along with apoptosis factors following *ex vivo* re-stimulation.

### Assessment of CD8^+^ T cell proliferation

We performed BrdU pulse treatment (0.8mg/ml drinking water, DW) for twelve days. Cells were surface-stained followed by intracellular staining for cytokines using the BD Perm/Fix kit. Later, cells were subjected to anti-BrdU antibody staining using a BrdU kit according to manufacturer’s instructions (BD Pharmingen). BrdU incorporation in DNA of proliferating cells was analyzed by flow cytometry.

### Bcl-2 inhibitor treatment

Bcl-2 specific inhibitor, ABT-199, was purchased from APExBIO (Houston, TX) and was re-suspended in 60% Phosal 50G, 30% Polyethylene Glycol 400 and 10% ethanol as described[65]. The inhibitor was administered (20 mg/kg body weight) daily by oral gavage for 10 days. On day 11, tissues were harvested and CD8^+^ T cells were analyzed by flow cytometry.

### HIF-1α inhibitor and HIF-1α agonist treatment

HIF-1α inhibitor, Echinomycin, was purchased from Cayman Chemical Company and re-suspended in 100% methanol. Echinomycin was administered intraperitoneally at the concentration of 20–30 μg/kg body weight in sterile 1X PBS every other day. A total of 5 doses were administered during the expansion and memory phases over 10 days, unless indicated. The HIF-1α agonist, Mimosine, was purchased from Sigma Aldrich and used at the concentration of 70 mg/kg body weight by s/c route every other day for 14 days during memory phase.

### Bone marrow chimera experiments

Bone marrow cells from congenic donor mice (WT/Thy1.1^+^ and CD4^Cre-HIF-1αfl/fl^/Ly5.2^+^) were transferred into lethally irradiated Ly5.1^+^ recipient mice. For generation of mixed-bone marrow chimera mice, the donor cells were mixed in 1:1 ratio and were co-transferred into Ly5.1^+^ recipient mice. After 2 months rest, mice were vaccinated to assess the role of HIF-1α in CD8^+^ T cells for expansion and generation of memory Tc17 cells.

### Statistical analysis

All statistical analysis was performed using a two-tailed unpaired Student t test except for analysis of fungal CFUs, which was measured by the non-parametric Kruskall-Wallis (one-way ANOVA). Prism 5 (GraphPad Software, Inc.) software was used to analyze all statistics. A two-tailed P value of ≤0.05 was considered statistically significant.

## Supporting information

S1 Fig Long-term persistence of memory Tc17 cells. Naïve IL17a^Cre^R26R^eYFP^ mice were vaccinated and draining LNs (dLNs) and spleens collected. Cells were stained and analyzed by flow cytometry. Percent of IL-17A producing cells among CD8^+^ eYFP^+^ T cells is shown on day 19 post-vaccination. **(B & C)** IL17a^Cre^R26R^eYFP^ mice were vaccinated and rested as in Fig 1. On indicated days, dLNs were harvested to enumerate CD8^+^ eYFP^+^ cells in vaccinated mice (**B**) or in donor T-cell recipient mice (**C**). N = 3–5 mice per group. Data is representative of at least two independent experiments. Mice were injected with GK1.5 throughout the experiment to deplete CD4^+^ T cells.
(TIF)

S2 Fig In vitro plasticity of memory Tc17 cells. Splenocytes from vaccinated IL17a^Cre^R26R^eYFP^ mice (D162 post-vaccination) were incubated with IL-12/IFNγ (10ng/ml) for 18 hrs in the presence of IL-2 (10ng/ml). Cells were washed and re-stimulated with anti-CD3/CD28 antibodies for 5 hrs before intracellular cytokine staining. **A.** Percent IFNγ cytokine-producing cells among activated Tc1 cells. **B.** Percent IL-17A and IFNγ cytokine-producing cells among activated eYFP^+^ Tc17 cells. Each respective colored line represents data from a single mouse. * p ≤ 0.05.
(TIF)

S3 Fig In vivo plasticity of memory Tc17 cells. Naïve IL17a^Cre^R26R^eYFP^ mice were vaccinated and rested for at least 46 days. Spleens were harvested and surface-stained for CD8^+^ T-cell markers along with PD-1 (**A**), intracellularly stained for FoxP3 and IL-22 (**B**) and stained for surface IL-1R1 and IL-23R followed by intracellular Stat3 (**C**). Frequency of IL-1R1 and IL-21 CD8^+^ T cells (**D**). Numbers represent frequencies among CD8^+^eYFP^+^/eYFP^-^ T cells. Histogram values represent mean florescence intensity. N = 4–5 mice. Data is representative of two independent experiments.
(TIF)

S4 Fig Phenotypic attributes of memory Tc17 cells. Naïve IL17a^Cre^R26R^eYFP^ mice were vaccinated and rested as described in Fig 6. Spleens were harvested and surface-stained for phenotypic markers on CD8^+^eYFP^+^ T cells. Numbers represent frequencies (mean ± SD) among CD8^+^ T cells. N = 5 mice/group. *P≤0.05.
(TIF)

S5 Fig Proliferative renewal of Tc17 cells. Naïve IL17a^Cre^R26R^eYFP^ mice were vaccinated, rested and pulsed with BrdU as in Fig 7. dLN cells were harvested on indicated days. Cells were surface-stained, intracellularly stained for cytokines, and stained with anti-BrdU. Numbers represent percent ± SD of BrdU+ cells among CD8^+^ CD44^hi^ T cells. N = 4–5 mice/group. **P≤0.01 and ****P≤0.0001.
(TIF)

S6 Fig Apoptosis of memory Tc17 cells. Naïve IL17a^Cre^R26R^eYFP^ mice were vaccinated and rested for 76 days as described in Fig 7B. Splenocytes were re-stimulated with anti-CD3 and -CD28 antibodies followed by staining for surface markers and intracellular staining for active-Caspase 3 and 8 molecules. Data represent dot plots gated on CD8^+^ T cells (top panels). Isotype control staining is shown (bottom).
(TIF)

S7 Fig Role of Bcl-2 for memory Tc17 cells. IL17a^Cre^R26R^eYFP^ mice were vaccinated, rested, treated with Bcl-2 inhibitor ABT-199 and tissues were harvested for analysis as described in **Fig 7**. (**A**) Frequency and total numbers of CD8^+^ T cells, activated and naïve CD8^+^ T cells in the tissues. (**B**) To assess proliferation, cells were stained with anti-Ki-67 mAb intracellularly following intracellular cytokine staining, and the frequencies of Ki-67^+^ cells were analyzed by flow cytometry. N = 4–5 mice/group. CD4^+^ T cells were depleted throughout the experiment. *P≤0.05 and **P≤0.01.
(TIF)

S8 Fig Impact of HIF-1α on memory Tc17 and Tc1 cells. Naïve IL17a^Cre^R26R^eYFP^ mice were vaccinated and rested as described in Fig 8. Splenocytes were harvested and surface-stained followed by intracellular staining for HIF-1α either directly *ex vivo* (**A**) or after re-stimulation with anti-CD3 and -CD28 antibodies (**B**). Histograms represent the mean florescence intensity of HIF-1α on different populations along with isotype control. (**C**) Mice were vaccinated, rested, and treated with either Echinomycin or vehicle as described in Fig 7. (**D**) Percent cytokine-producing cells among CD8^+^CD44^hi^ eYFP^+^ T cells. Numbers are percent ± SD of eYFP^+^ among total splenocytes or CD8^+^ T cells (parenthesis). N = 4–5 mice/group.
(TIF)

## References

[1] Seder RA, Ahmed R (2003) Similarities and differences in CD4+ and CD8+ effector and memory T cell generation. Nat Immunol 4: 835–842. doi: 10.1038/ni969 12942084 10.1038/ni969

[2] Miller JD, van der Most RG, Akondy RS, Glidewell JT, Albott S, et al (2008) Human effector and memory CD8+ T cell responses to smallpox and yellow fever vaccines. Immunity 28: 710–722. doi: 10.1016/j.immuni.2008.02.020 18468462 10.1016/j.immuni.2008.02.020

[3] Murali-Krishna K, Lau LL, Sambhara S, Lemonnier F, Altman J, et al (1999) Persistence of memory CD8 T cells in MHC class I-deficient mice. Science 286: 1377–1381. 10558996 10.1126/science.286.5443.1377

[4] Hammarlund E, Lewis MW, Hansen SG, Strelow LI, Nelson JA, et al (2003) Duration of antiviral immunity after smallpox vaccination. Nat Med 9: 1131–1137. doi: 10.1038/nm917 12925846 10.1038/nm917

[5] Surh CD, Sprent J (2008) Homeostasis of naive and memory T cells. Immunity 29: 848–862. doi: 10.1016/j.immuni.2008.11.002 19100699 10.1016/j.immuni.2008.11.002

[6] Ku CC, Murakami M, Sakamoto A, Kappler J, Marrack P (2000) Control of homeostasis of CD8+ memory T cells by opposing cytokines. Science 288: 675–678. 10784451 10.1126/science.288.5466.675

[7] Schluns KS, Kieper WC, Jameson SC, Lefrancois L (2000) Interleukin-7 mediates the homeostasis of naive and memory CD8 T cells in vivo. Nat Immunol 1: 426–432. doi: 10.1038/80868 11062503 10.1038/80868

[8] Kaech SM, Tan JT, Wherry EJ, Konieczny BT, Surh CD, et al (2003) Selective expression of the interleukin 7 receptor identifies effector CD8 T cells that give rise to long-lived memory cells. Nat Immunol 4: 1191–1198. doi: 10.1038/ni1009 14625547 10.1038/ni1009

[9] Kaech SM, Cui W (2012) Transcriptional control of effector and memory CD8+ T cell differentiation. Nat Rev Immunol 12: 749–761. doi: 10.1038/nri3307 23080391 10.1038/nri3307 PMC4137483

[10] Lefrancois L, Marzo AL (2006) The descent of memory T-cell subsets. Nat Rev Immunol 6: 618–623. doi: 10.1038/nri1866 16868553 10.1038/nri1866

[11] Haines CJ, Chen Y, Blumenschein WM, Jain R, Chang C, et al (2013) Autoimmune memory T helper 17 cell function and expansion are dependent on interleukin-23. Cell Rep 3: 1378–1388. doi: 10.1016/j.celrep.2013.03.035 23623497 10.1016/j.celrep.2013.03.035

[12] Kryczek I, Zhao E, Liu Y, Wang Y, Vatan L, et al (2011) Human TH17 cells are long-lived effector memory cells. Sci Transl Med 3: 104ra100 doi: 10.1126/scitranslmed.3002949 21998407 10.1126/scitranslmed.3002949 PMC3345568

[13] Muranski P, Borman ZA, Kerkar SP, Klebanoff CA, Ji Y, et al (2011) Th17 cells are long lived and retain a stem cell-like molecular signature. Immunity 35: 972–985. doi: 10.1016/j.immuni.2011.09.019 22177921 10.1016/j.immuni.2011.09.019 PMC3246082

[14] Martin-Orozco N, Muranski P, Chung Y, Yang XO, Yamazaki T, et al (2009) T helper 17 cells promote cytotoxic T cell activation in tumor immunity. Immunity 31: 787–798. doi: 10.1016/j.immuni.2009.09.014 19879162 10.1016/j.immuni.2009.09.014 PMC2787786

[15] Pepper M, Linehan JL, Pagan AJ, Zell T, Dileepan T, et al (2010) Different routes of bacterial infection induce long-lived TH1 memory cells and short-lived TH17 cells. Nat Immunol 11: 83–89. doi: 10.1038/ni.1826 19935657 10.1038/ni.1826 PMC2795784

[16] Lindenstrom T, Woodworth J, Dietrich J, Aagaard C, Andersen P, et al (2012) Vaccine-induced th17 cells are maintained long-term postvaccination as a distinct and phenotypically stable memory subset. Infect Immun 80: 3533–3544. doi: 10.1128/IAI.00550-12 22851756 10.1128/IAI.00550-12 PMC3457559

[17] Hirota K, Duarte JH, Veldhoen M, Hornsby E, Li Y, et al (2011) Fate mapping of IL-17-producing T cells in inflammatory responses. Nat Immunol 12: 255–263. doi: 10.1038/ni.1993 21278737 10.1038/ni.1993 PMC3040235

[18] Hernandez-Santos N, Huppler AR, Peterson AC, Khader SA, McKenna KC, et al (2013) Th17 cells confer long-term adaptive immunity to oral mucosal Candida albicans infections. Mucosal Immunol 6: 900–910. doi: 10.1038/mi.2012.128 23250275 10.1038/mi.2012.128 PMC3608691

[19] Miossec P, Kolls JK (2012) Targeting IL-17 and TH17 cells in chronic inflammation. Nat Rev Drug Discov 11: 763–776. doi: 10.1038/nrd3794 23023676 10.1038/nrd3794

[20] Yang BH, Floess S, Hagemann S, Deyneko IV, Groebe L, et al (2015) Development of a unique epigenetic signature during in vivo Th17 differentiation. Nucleic Acids Res 43: 1537–1548. doi: 10.1093/nar/gkv014 25593324 10.1093/nar/gkv014 PMC4330377

[21] Mukasa R, Balasubramani A, Lee YK, Whitley SK, Weaver BT, et al (2010) Epigenetic instability of cytokine and transcription factor gene loci underlies plasticity of the T helper 17 cell lineage. Immunity 32: 616–627. doi: 10.1016/j.immuni.2010.04.016 20471290 10.1016/j.immuni.2010.04.016 PMC3129685

[22] Lee YK, Turner H, Maynard CL, Oliver JR, Chen D, et al (2009) Late developmental plasticity in the T helper 17 lineage. Immunity 30: 92–107. doi: 10.1016/j.immuni.2008.11.005 19119024 10.1016/j.immuni.2008.11.005 PMC3607320

[23] Muranski P, Restifo NP (2013) Essentials of Th17 cell commitment and plasticity. Blood 121: 2402–2414. doi: 10.1182/blood-2012-09-378653 23325835 10.1182/blood-2012-09-378653 PMC3612853

[24] Nurieva R, Yang XXO, Chung Y, Dong C (2009) Cutting Edge: In Vitro Generated Th17 Cells Maintain Their Cytokine Expression Program in Normal but Not Lymphopenic Hosts. Journal of Immunology 182: 2565–2568. 10.4049/jimmunol.0803931 PMC2755098 19234148

[25] Duhen R, Glatigny S, Arbelaez CA, Blair TC, Oukka M, et al (2013) Cutting edge: the pathogenicity of IFN-gamma-producing Th17 cells is independent of T-bet. J Immunol 190: 4478–4482. doi: 10.4049/jimmunol.1203172 23543757 10.4049/jimmunol.1203172 PMC3633668

[26] El-Behi M, Ciric B, Dai H, Yan Y, Cullimore M, et al (2011) The encephalitogenicity of T(H)17 cells is dependent on IL-1- and IL-23-induced production of the cytokine GM-CSF. Nat Immunol 12: 568–575. doi: 10.1038/ni.2031 21516111 10.1038/ni.2031 PMC3116521

[27] Zielinski CE, Mele F, Aschenbrenner D, Jarrossay D, Ronchi F, et al (2012) Pathogen-induced human TH17 cells produce IFN-gamma or IL-10 and are regulated by IL-1beta. Nature 484: 514–518. doi: 10.1038/nature10957 22466287 10.1038/nature10957

[28] Liang Y, Pan HF, Ye DQ (2015) Tc17 Cells in Immunity and Systemic Autoimmunity. Int Rev Immunol 34: 318–331. doi: 10.3109/08830185.2014.954698 25259411 10.3109/08830185.2014.954698

[29] Hamada H, Garcia-Hernandez Mde L, Reome JB, Misra SK, Strutt TM, et al (2009) Tc17, a unique subset of CD8 T cells that can protect against lethal influenza challenge. J Immunol 182: 3469–3481. doi: 10.4049/jimmunol.0801814 19265125 10.4049/jimmunol.0801814 PMC2667713

[30] Nanjappa SG, Heninger E, Wuthrich M, Gasper DJ, Klein BS (2012) Tc17 cells mediate vaccine immunity against lethal fungal pneumonia in immune deficient hosts lacking CD4+ T cells. PLoS Pathog 8: e1002771 doi: 10.1371/journal.ppat.1002771 22829762 10.1371/journal.ppat.1002771 PMC3400565

[31] Yeh N, Glosson NL, Wang N, Guindon L, McKinley C, et al (2010) Tc17 cells are capable of mediating immunity to vaccinia virus by acquisition of a cytotoxic phenotype. J Immunol 185: 2089–2098. doi: 10.4049/jimmunol.1000818 20624947 10.4049/jimmunol.1000818 PMC2916954

[32] Huber M, Heink S, Pagenstecher A, Reinhard K, Ritter J, et al (2013) IL-17A secretion by CD8+ T cells supports Th17-mediated autoimmune encephalomyelitis. J Clin Invest 123: 247–260. doi: 10.1172/JCI63681 23221338 10.1172/JCI63681 PMC3533283

[33] Tajima M, Wakita D, Satoh T, Kitamura H, Nishimura T (2011) IL-17/IFN-gamma double producing CD8+ T (Tc17/IFN-gamma) cells: a novel cytotoxic T-cell subset converted from Tc17 cells by IL-12. Int Immunol 23: 751–759. doi: 10.1093/intimm/dxr086 22039016 10.1093/intimm/dxr086

[34] Gartlan KH, Markey KA, Varelias A, Bunting MD, Koyama M, et al (2015) Tc17 cells are a proinflammatory, plastic lineage of pathogenic CD8+ T cells that induce GVHD without antileukemic effects. Blood 126: 1609–1620. doi: 10.1182/blood-2015-01-622662 26206951 10.1182/blood-2015-01-622662

[35] Brown GD, Denning DW, Gow NA, Levitz SM, Netea MG, et al (2012) Hidden killers: human fungal infections. Sci Transl Med 4: 165rv113. 10.1126/scitranslmed.3004404 23253612

[36] Nanjappa SG, Heninger E, Wuthrich M, Sullivan T, Klein B (2012) Protective antifungal memory CD8(+) T cells are maintained in the absence of CD4(+) T cell help and cognate antigen in mice. J Clin Invest 122: 987–999. doi: 10.1172/JCI58762 22354169 10.1172/JCI58762 PMC3287218

[37] Patel DD, Kuchroo VK (2015) Th17 Cell Pathway in Human Immunity: Lessons from Genetics and Therapeutic Interventions. Immunity 43: 1040–1051. doi: 10.1016/j.immuni.2015.12.003 26682981 10.1016/j.immuni.2015.12.003

[38] Wuthrich M, Gern B, Hung CY, Ersland K, Rocco N, et al (2011) Vaccine-induced protection against 3 systemic mycoses endemic to North America requires Th17 cells in mice. J Clin Invest 121: 554–568. doi: 10.1172/JCI43984 21206087 10.1172/JCI43984 PMC3026727

[39] Nanjappa SG, Hernandez-Santos N, Galles K, Wuthrich M, Suresh M, et al (2015) Intrinsic MyD88-Akt1-mTOR Signaling Coordinates Disparate Tc17 and Tc1 Responses during Vaccine Immunity against Fungal Pneumonia. PLoS Pathog 11: e1005161 doi: 10.1371/journal.ppat.1005161 26367276 10.1371/journal.ppat.1005161 PMC4569330

[40] Wuthrich M, Filutowicz HI, Warner T, Deepe GS Jr., Klein BS (2003) Vaccine immunity to pathogenic fungi overcomes the requirement for CD4 help in exogenous antigen presentation to CD8+ T cells: implications for vaccine development in immune-deficient hosts. J Exp Med 197: 1405–1416. doi: 10.1084/jem.20030109 12782709 10.1084/jem.20030109 PMC2193905

[41] Wei L, Laurence A, Elias KM, O'Shea JJ (2007) IL-21 is produced by Th17 cells and drives IL-17 production in a STAT3-dependent manner. Journal of Biological Chemistry 282: 34605–34610. doi: 10.1074/jbc.M705100200 17884812 10.1074/jbc.M705100200 PMC2323680

[42] Hanninen A, Maksimow M, Alam C, Morgan DJ, Jalkanen S (2011) Ly6C supports preferential homing of central memory CD8+ T cells into lymph nodes. Eur J Immunol 41: 634–644. doi: 10.1002/eji.201040760 21308682 10.1002/eji.201040760

[43] Joshi NS, Kaech SM (2008) Effector CD8 T cell development: a balancing act between memory cell potential and terminal differentiation. J Immunol 180: 1309–1315. 18209024 10.4049/jimmunol.180.3.1309

[44] Coquet JM, Middendorp S, van der Horst G, Kind J, Veraar EA, et al (2013) The CD27 and CD70 costimulatory pathway inhibits effector function of T helper 17 cells and attenuates associated autoimmunity. Immunity 38: 53–65. doi: 10.1016/j.immuni.2012.09.009 23159439 10.1016/j.immuni.2012.09.009

[45] Mackay LK, Rahimpour A, Ma JZ, Collins N, Stock AT, et al (2013) The developmental pathway for CD103(+)CD8+ tissue-resident memory T cells of skin. Nat Immunol 14: 1294–1301. doi: 10.1038/ni.2744 24162776 10.1038/ni.2744

[46] Boudousquie C, Danilo M, Pousse L, Jeevan-Raj B, Angelov GS, et al (2014) Differences in the transduction of canonical Wnt signals demarcate effector and memory CD8 T cells with distinct recall proliferation capacity. J Immunol 193: 2784–2791. doi: 10.4049/jimmunol.1400465 25127860 10.4049/jimmunol.1400465

[47] McGeachy MJ (2013) Th17 memory cells: live long and proliferate. J Leukoc Biol 94: 921–926. doi: 10.1189/jlb.0313113 24006508 10.1189/jlb.0313113

[48] Boyman O, Letourneau S, Krieg C, Sprent J (2009) Homeostatic proliferation and survival of naive and memory T cells. Eur J Immunol 39: 2088–2094. doi: 10.1002/eji.200939444 19637200 10.1002/eji.200939444

[49] Wojciechowski S, Tripathi P, Bourdeau T, Acero L, Grimes HL, et al (2007) Bim/Bcl-2 balance is critical for maintaining naive and memory T cell homeostasis. J Exp Med 204: 1665–1675. doi: 10.1084/jem.20070618 17591857 10.1084/jem.20070618 PMC2118628

[50] Kurtulus S, Tripathi P, Moreno-Fernandez ME, Sholl A, Katz JD, et al (2011) Bcl-2 allows effector and memory CD8+ T cells to tolerate higher expression of Bim. J Immunol 186: 5729–5737. doi: 10.4049/jimmunol.1100102 21451108 10.4049/jimmunol.1100102 PMC4222684

[51] Shi LZ, Wang R, Huang G, Vogel P, Neale G, et al (2011) HIF1alpha-dependent glycolytic pathway orchestrates a metabolic checkpoint for the differentiation of TH17 and Treg cells. J Exp Med 208: 1367–1376. doi: 10.1084/jem.20110278 21708926 10.1084/jem.20110278 PMC3135370

[52] Fan D, Coughlin LA, Neubauer MM, Kim J, Kim MS, et al (2015) Activation of HIF-1alpha and LL-37 by commensal bacteria inhibits Candida albicans colonization. Nat Med 21: 808–814. doi: 10.1038/nm.3871 26053625 10.1038/nm.3871 PMC4496259

[53] Croft M (2014) The TNF family in T cell differentiation and function—unanswered questions and future directions. Semin Immunol 26: 183–190. doi: 10.1016/j.smim.2014.02.005 24613728 10.1016/j.smim.2014.02.005 PMC4099277

[54] Jain R, Chen Y, Kanno Y, Joyce-Shaikh B, Vahedi G, et al (2016) Interleukin-23-Induced Transcription Factor Blimp-1 Promotes Pathogenicity of T Helper 17 Cells. Immunity 44: 131–142. doi: 10.1016/j.immuni.2015.11.009 26750311 10.1016/j.immuni.2015.11.009 PMC11608061

[55] Murali-Krishna K, Ahmed R (2000) Cutting edge: Naive T cells masquerading as memory cells. Journal of Immunology 165: 1733–1737. 10.4049/jimmunol.165.4.1733 10925249

[56] Bachmann MF, Beerli RR, Agnellini P, Wolint P, Schwarz K, et al (2006) Long-lived memory CD8(+) T cells are programmed by prolonged antigen exposure and low levels of cellular activation. European Journal of Immunology 36: 842–854. doi: 10.1002/eji.200535730 16552716 10.1002/eji.200535730

[57] Schluns KS, Lefrancois L (2003) Cytokine control of memory T-cell development and survival. Nat Rev Immunol 3: 269–279. doi: 10.1038/nri1052 12669018 10.1038/nri1052

[58] Aiello FB, Graciotti L, Procopio AD, Keller JR, Durum SK (2013) Stemness of T cells and the hematopoietic stem cells: fate, memory, niche, cytokines. Cytokine Growth Factor Rev 24: 485–501. doi: 10.1016/j.cytogfr.2013.10.002 24231048 10.1016/j.cytogfr.2013.10.002 PMC6390295

[59] Palazon A, Goldrath AW, Nizet V, Johnson RS (2014) HIF transcription factors, inflammation, and immunity. Immunity 41: 518–528. doi: 10.1016/j.immuni.2014.09.008 25367569 10.1016/j.immuni.2014.09.008 PMC4346319

[60] Doedens AL, Phan AT, Stradner MH, Fujimoto JK, Nguyen JV, et al (2013) Hypoxia-inducible factors enhance the effector responses of CD8(+) T cells to persistent antigen. Nat Immunol 14: 1173–1182. doi: 10.1038/ni.2714 24076634 10.1038/ni.2714 PMC3977965

[61] Intlekofer AM, Banerjee A, Takemoto N, Gordon SM, Dejong CS, et al (2008) Anomalous type 17 response to viral infection by CD8+ T cells lacking T-bet and eomesodermin. Science 321: 408–411. doi: 10.1126/science.1159806 18635804 10.1126/science.1159806 PMC2807624

[62] Dang EV, Barbi J, Yang HY, Jinasena D, Yu H, et al (2011) Control of T(H)17/T(reg) balance by hypoxia-inducible factor 1. Cell 146: 772–784. doi: 10.1016/j.cell.2011.07.033 21871655 10.1016/j.cell.2011.07.033 PMC3387678

[63] Finlay DK, Rosenzweig E, Sinclair LV, Feijoo-Carnero C, Hukelmann JL, et al (2012) PDK1 regulation of mTOR and hypoxia-inducible factor 1 integrate metabolism and migration of CD8+ T cells. J Exp Med 209: 2441–2453. doi: 10.1084/jem.20112607 23183047 10.1084/jem.20112607 PMC3526360

[64] Chang JT, Wherry EJ, Goldrath AW (2014) Molecular regulation of effector and memory T cell differentiation. Nat Immunol 15: 1104–1115. doi: 10.1038/ni.3031 25396352 10.1038/ni.3031 PMC4386685

[65] Souers AJ, Leverson JD, Boghaert ER, Ackler SL, Catron ND, et al (2013) ABT-199, a potent and selective BCL-2 inhibitor, achieves antitumor activity while sparing platelets. Nature Medicine 19: 202–208. doi: 10.1038/nm.3048 23291630 10.1038/nm.3048

