# Supplementary figures and images for: Antifungal Tc17 cells are durable and stable, persisting as long-lasting vaccine memory without plasticity towards IFNγ cells
Source: PLoS Pathog. 2017 May 22;13(5):e1006356. doi: 10.1371/journal.ppat.1006356 (PMC5456400; doi:10.1371/journal.ppat.1006356)

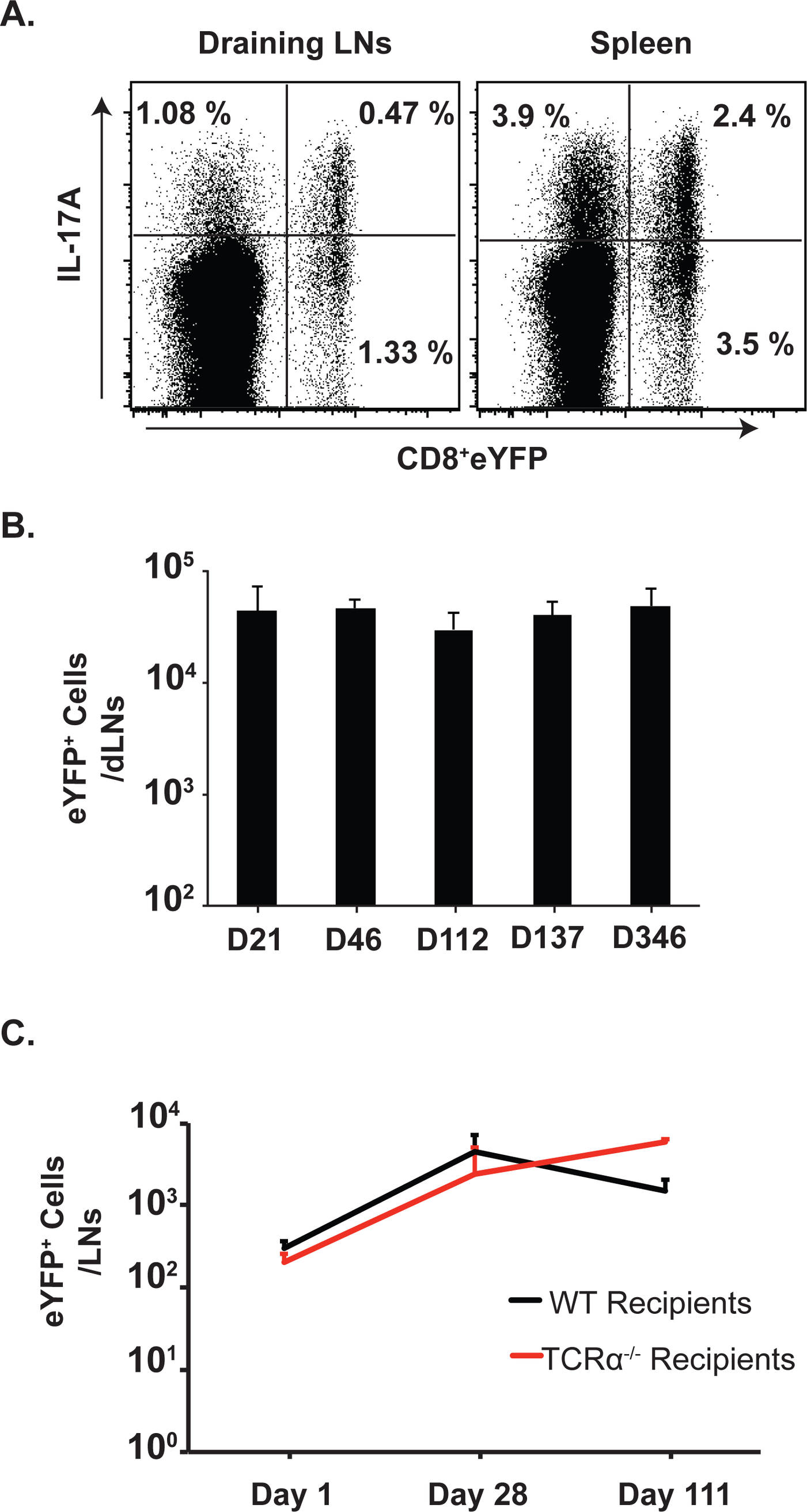

Supplement: S1 Fig — Naïve IL17aCreR26ReYFP mice were vaccinated and draining LNs (dLNs) and spleens collected. Cells were stained and analyzed by flow cytometry. Percent of IL-17A producing cells among CD8+ eYFP+ T cells is shown on day 19 post-vaccination. (B & C) IL17aCreR26ReYFP mice were vaccinated and rested as in Fig 1. On indicated days, dLNs were harvested to enumerate CD8+ eYFP+ cells in vaccinated mice (B) or in donor T-cell recipient mice (C). N = 3–5 mice per group. Data is representative of at least two independent experiments. Mice were injected with GK1.5 throughout the experiment to deplete CD4+ T cells. (TIF) [file ppat.1006356.s001.tif]

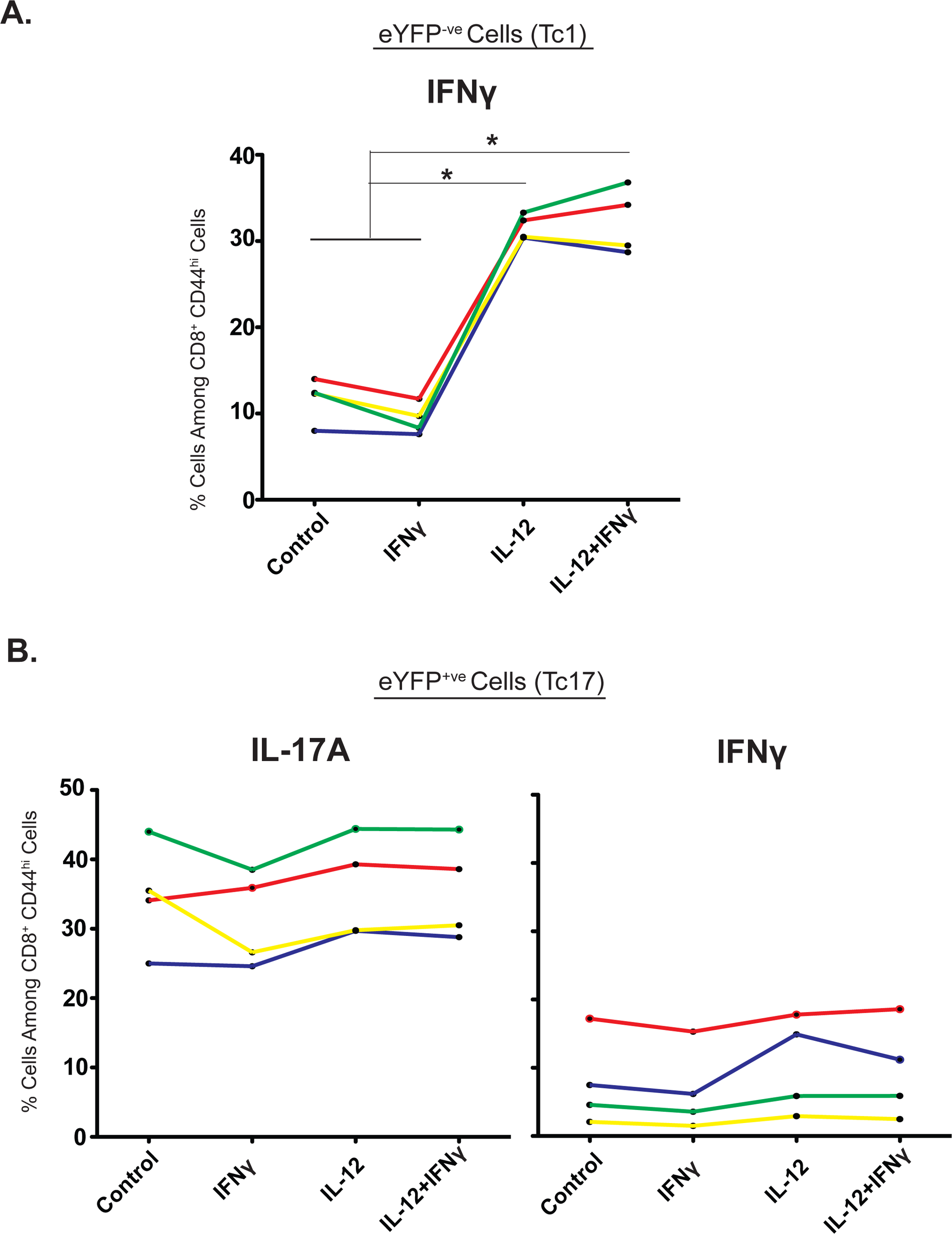

Supplement: S2 Fig — Splenocytes from vaccinated IL17aCreR26ReYFP mice (D162 post-vaccination) were incubated with IL-12/IFNγ (10ng/ml) for 18 hrs in the presence of IL-2 (10ng/ml). Cells were washed and re-stimulated with anti-CD3/CD28 antibodies for 5 hrs before intracellular cytokine staining. A. Percent IFNγ cytokine-producing cells among activated Tc1 cells. B. Percent IL-17A and IFNγ cytokine-producing cells among activated eYFP+ Tc17 cells. Each respective colored line represents data from a single mouse. * p ≤ 0.05. (TIF) [file ppat.1006356.s002.tif]

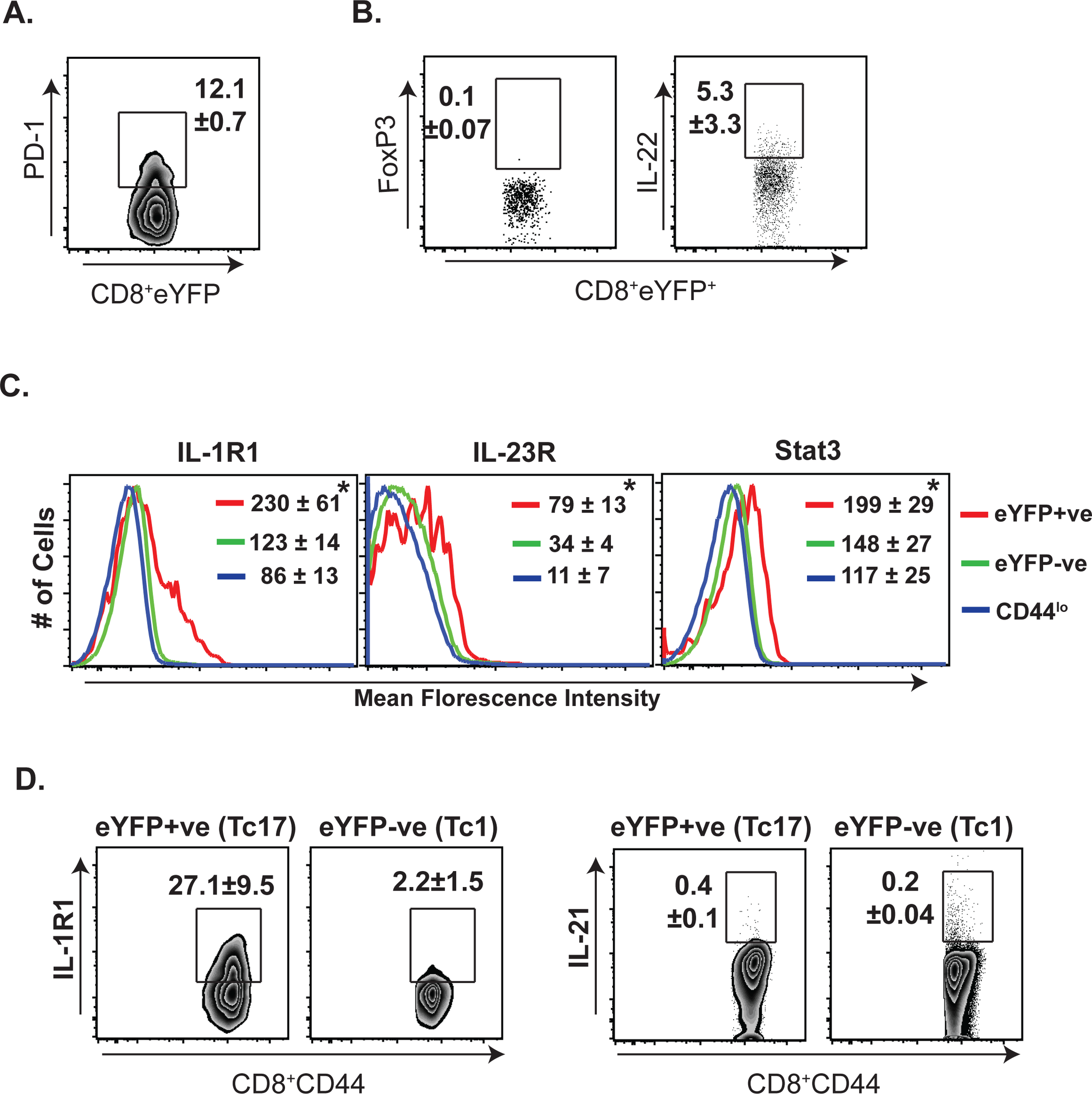

Supplement: S3 Fig — Naïve IL17aCreR26ReYFP mice were vaccinated and rested for at least 46 days. Spleens were harvested and surface-stained for CD8+ T-cell markers along with PD-1 (A), intracellularly stained for FoxP3 and IL-22 (B) and stained for surface IL-1R1 and IL-23R followed by intracellular Stat3 (C). Frequency of IL-1R1 and IL-21 CD8+ T cells (D). Numbers represent frequencies among CD8+eYFP+/eYFP- T cells. Histogram values represent mean florescence intensity. N = 4–5 mice. Data is representative of two independent experiments. (TIF) [file ppat.1006356.s003.tif]

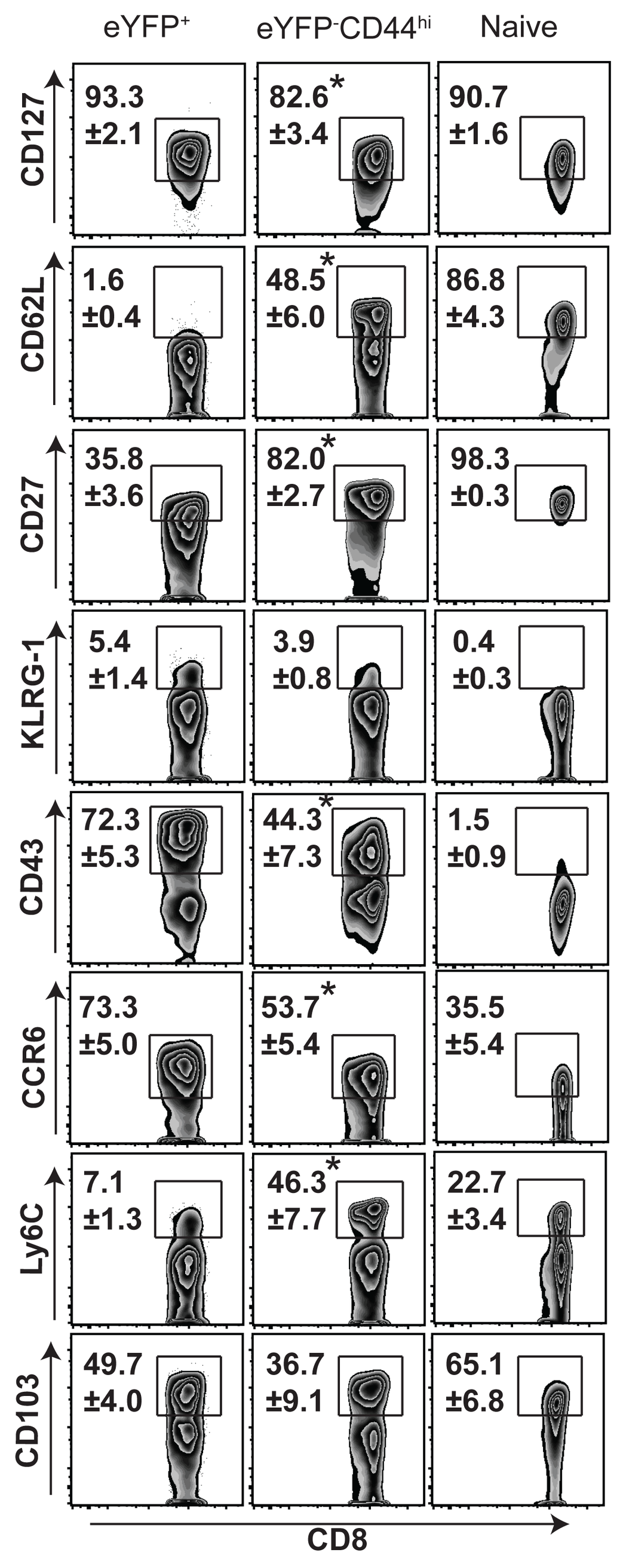

Supplement: S4 Fig — Naïve IL17aCreR26ReYFP mice were vaccinated and rested as described in Fig 6. Spleens were harvested and surface-stained for phenotypic markers on CD8+eYFP+ T cells. Numbers represent frequencies (mean ± SD) among CD8+ T cells. N = 5 mice/group. *P≤0.05. (TIF) [file ppat.1006356.s004.tif]

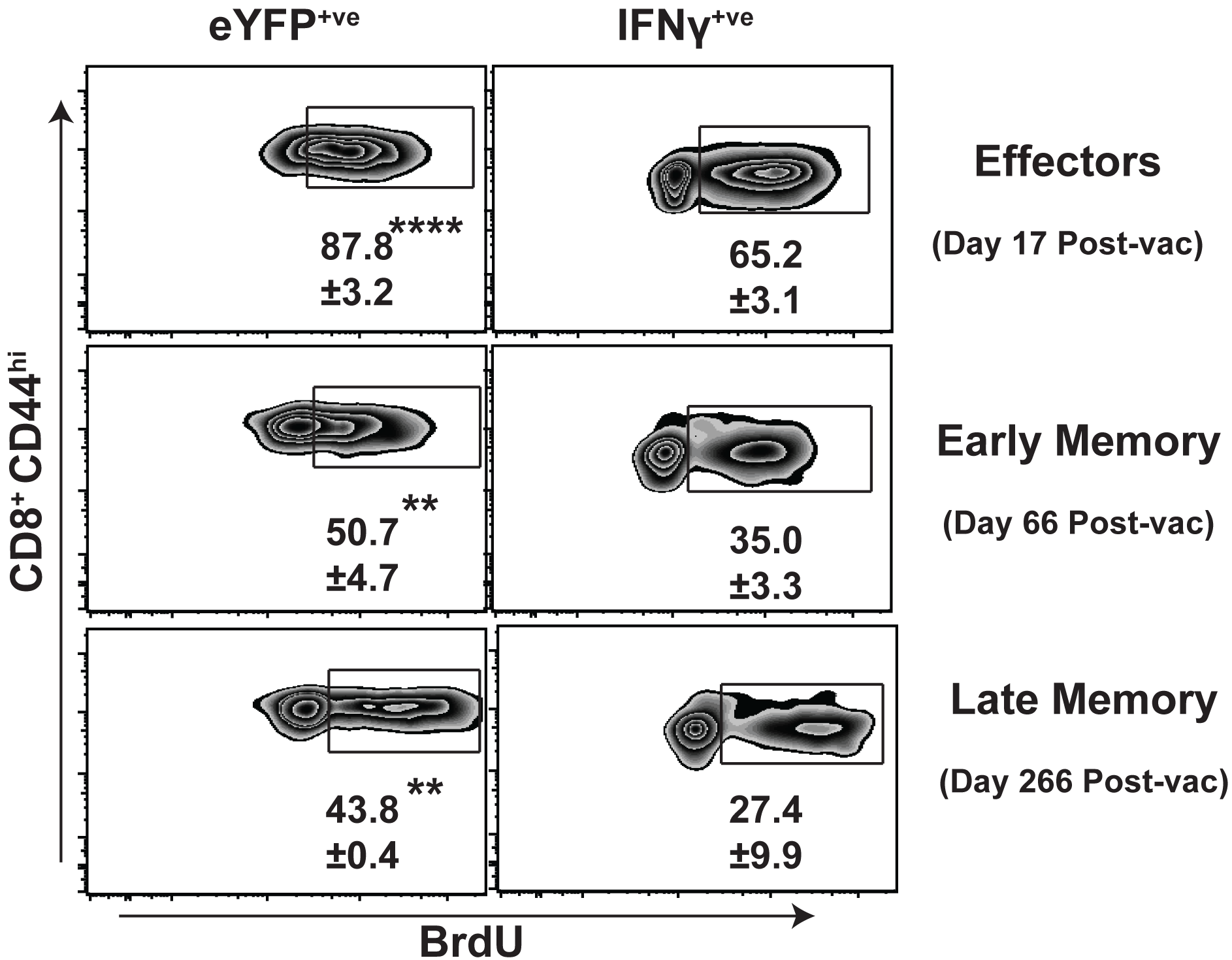

Supplement: S5 Fig — Naïve IL17aCreR26ReYFP mice were vaccinated, rested and pulsed with BrdU as in Fig 7. dLN cells were harvested on indicated days. Cells were surface-stained, intracellularly stained for cytokines, and stained with anti-BrdU. Numbers represent percent ± SD of BrdU+ cells among CD8+ CD44hi T cells. N = 4–5 mice/group. **P≤0.01 and ****P≤0.0001. (TIF) [file ppat.1006356.s005.tif]

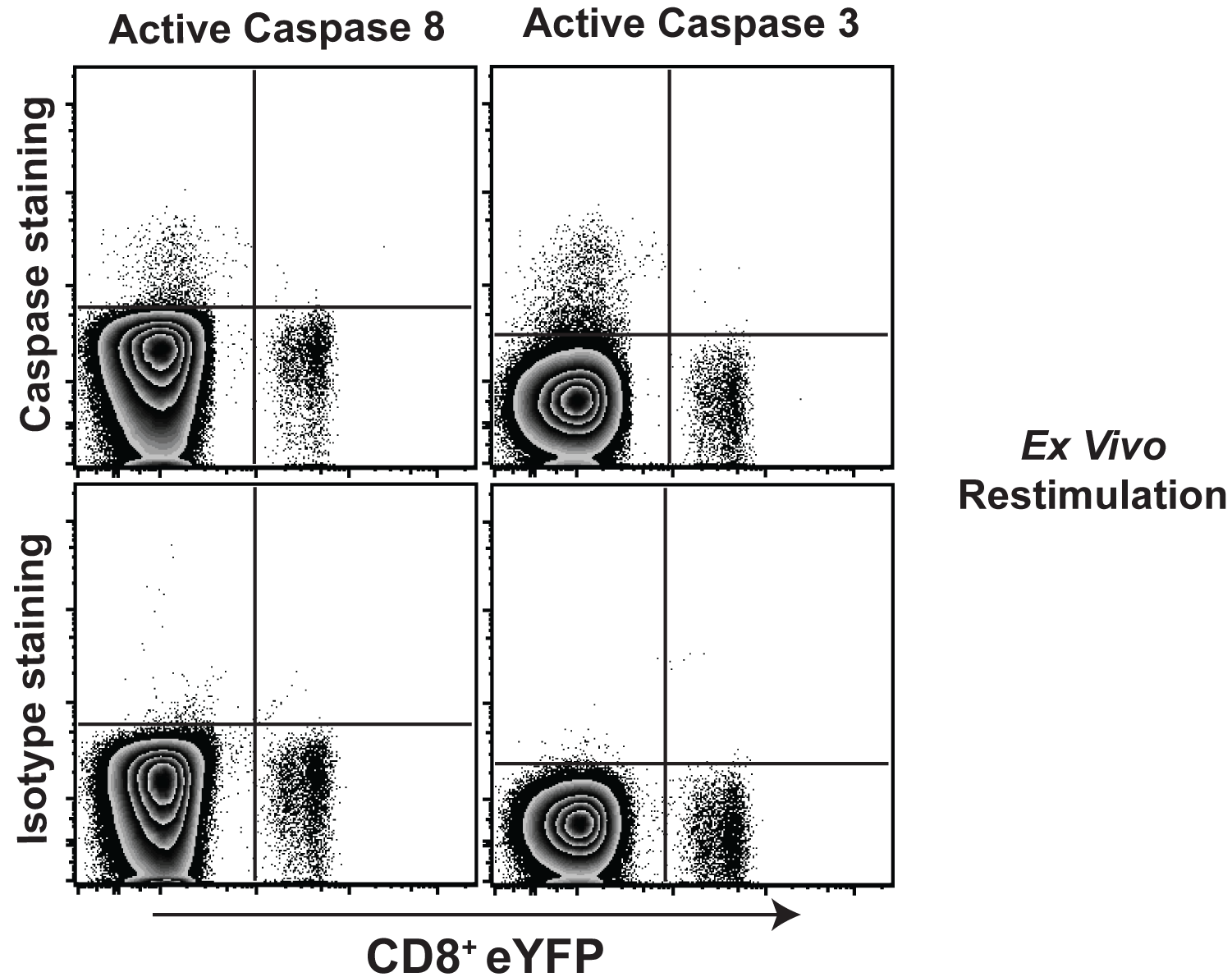

Supplement: S6 Fig — Naïve IL17aCreR26ReYFP mice were vaccinated and rested for 76 days as described in Fig 7B. Splenocytes were re-stimulated with anti-CD3 and -CD28 antibodies followed by staining for surface markers and intracellular staining for active-Caspase 3 and 8 molecules. Data represent dot plots gated on CD8+ T cells (top panels). Isotype control staining is shown (bottom). (TIF) [file ppat.1006356.s006.tif]

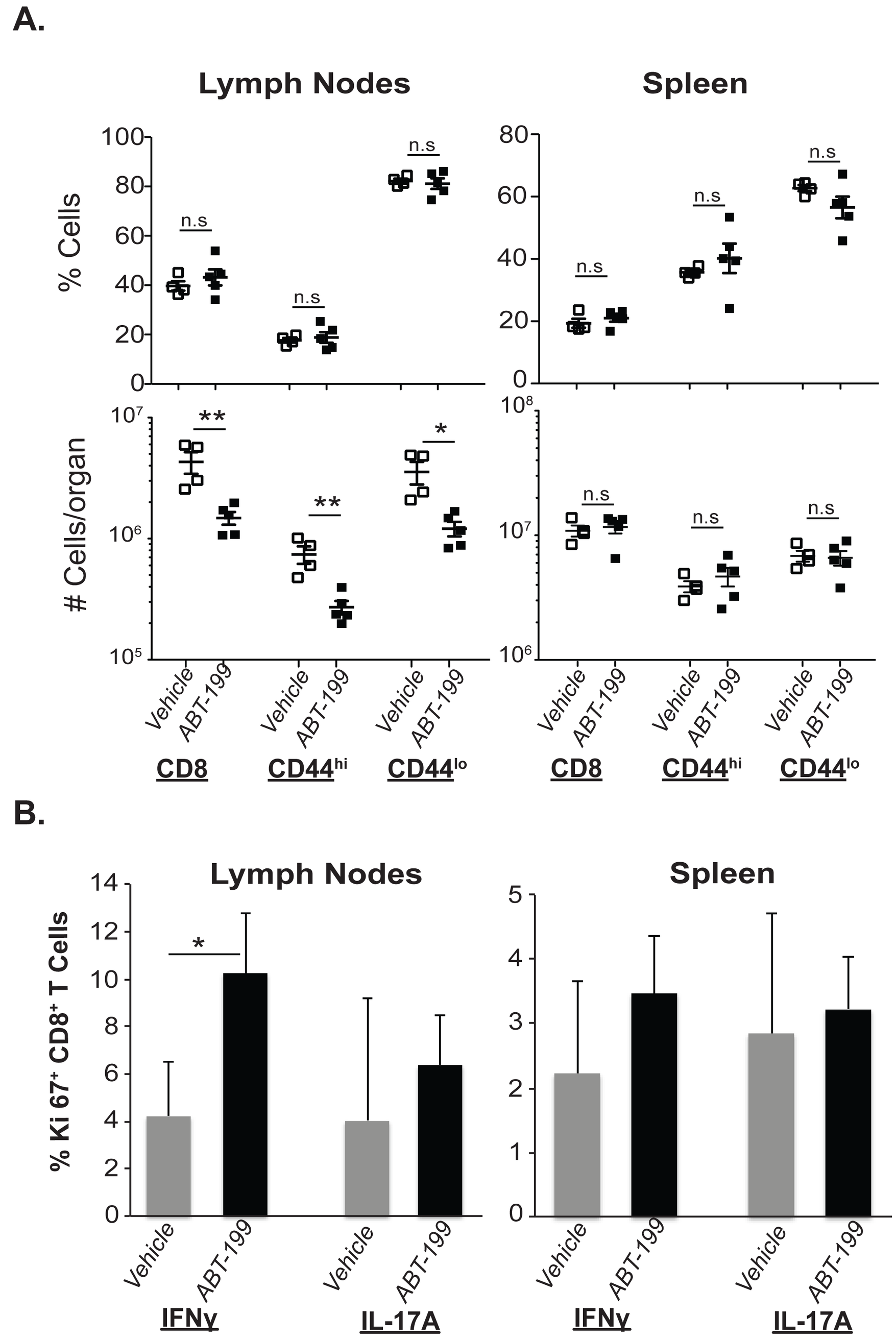

Supplement: S7 Fig — IL17aCreR26ReYFP mice were vaccinated, rested, treated with Bcl-2 inhibitor ABT-199 and tissues were harvested for analysis as described in Fig 7. (A) Frequency and total numbers of CD8+ T cells, activated and naïve CD8+ T cells in the tissues. (B) To assess proliferation, cells were stained with anti-Ki-67 mAb intracellularly following intracellular cytokine staining, and the frequencies of Ki-67+ cells were analyzed by flow cytometry. N = 4–5 mice/group. CD4+ T cells were depleted throughout the experiment. *P≤0.05 and **P≤0.01. (TIF) [file ppat.1006356.s007.tif]

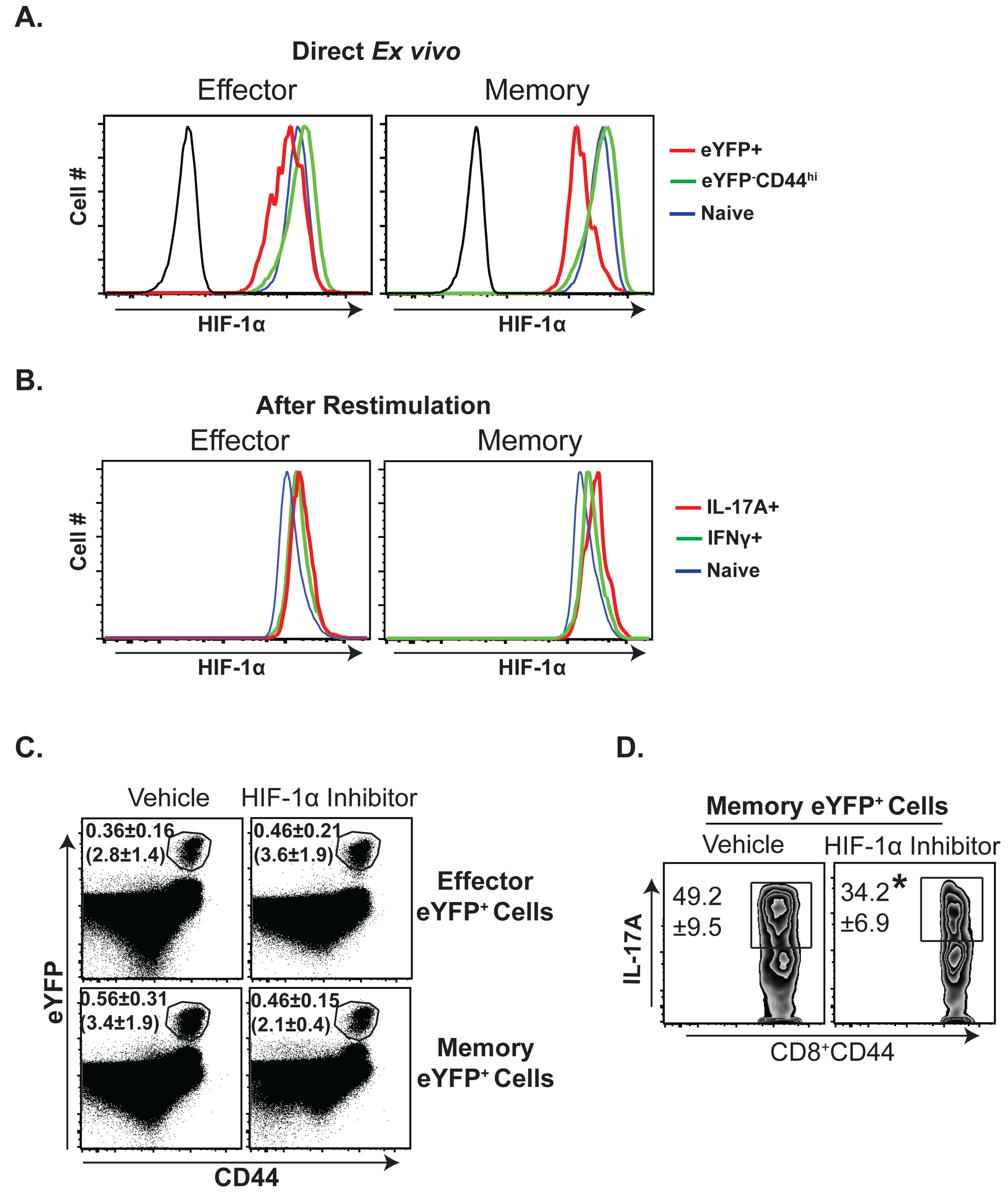

Supplement: S8 Fig — Naïve IL17aCreR26ReYFP mice were vaccinated and rested as described in Fig 8. Splenocytes were harvested and surface-stained followed by intracellular staining for HIF-1α either directly ex vivo (A) or after re-stimulation with anti-CD3 and -CD28 antibodies (B). Histograms represent the mean florescence intensity of HIF-1α on different populations along with isotype control. (C) Mice were vaccinated, rested, and treated with either Echinomycin or vehicle as described in Fig 7. (D) Percent cytokine-producing cells among CD8+CD44hi eYFP+ T cells. Numbers are percent ± SD of eYFP+ among total splenocytes or CD8+ T cells (parenthesis). N = 4–5 mice/group. (TIF) [file ppat.1006356.s008.tif]
